# Impact of scattering phase function and polarization on the accuracy of diffuse and sub-diffuse spatial frequency domain imaging

**DOI:** 10.1117/1.JBO.29.9.095001

**Published:** 2024-09-06

**Authors:** Alec B. Walter, E. Duco Jansen

**Affiliations:** a Vanderbilt University, Department of Biomedical Engineering, Nashville, Tennessee, United States; b Vanderbilt University, Biophotonics Center, Nashville, Tennessee, United States; c Vanderbilt University Medical Center, Department of Neurosurgery, Nashville, Tennessee, United States

**Keywords:** optical properties, scattering, spatial frequencies, backscattering, absorption, polarization

## Abstract

**Significance:**

Although spatial frequency domain imaging (SFDI) has been well characterized under diffuse optical conditions, tissue measurements made outside the diffuse regime can provide new diagnostic information. Before such measurements can become clinically relevant, however, the behavior of sub-diffuse SFDI and its effect on the accuracy of derived tissue parameters must be assessed.

**Aim:**

We aim to characterize the impact that both the assumed scattering phase function (SPF) and the polarization state of the illumination light source have on the accuracy of SFDI-derived optical properties when operating under diffuse or sub-diffuse conditions, respectively.

**Approach:**

Through the use of a set of well-characterized optical phantoms, SFDI accuracy was assessed at four wavelengths (395, 545, 625, and 850 nm) and two different spatial frequencies (0.3 and $1.0\;{\mathrm{mm}}^{-1}$), which provided a broad range of diffuse and sub-diffuse conditions, using three different SPFs. To determine the effects of polarization, the SFDI accuracy was assessed using both unpolarized and cross-polarized illumination.

**Results:**

It was found that the assumed SPF has a direct and significant impact on the accuracy of the SFDI-derived optical properties, with the best choice of SPF being dictated by the polarization state. As unpolarized SFDI retains the sub-diffuse portion of the signal, optical properties were found to be more accurate when using the full SPF that includes forward and backscattering components. By contrast, cross-polarized SFDI yielded accurate optical properties when using a forward-scattering SPF, matching the behavior of cross-polarization to attenuate the immediate backscattering of sub-diffuse reflectance. Using the correct pairings of SPF and polarization enabled using a reflectance standard, instead of a more subjective phantom, as the reference measurement.

**Conclusions:**

These results provide the foundation for a more thorough understanding of SFDI and enable new applications of this technology in which sub-diffuse conditions dominate (e.g., $\mu_{a}≮\mu_{s}^{\prime }$) or high spatial frequencies are required.

## 1 Introduction

Over the past decade, spatial frequency domain imaging (SFDI) has been widely adopted due to its unique capacity to provide quantitative information about endogenous tissue optical properties using diffuse imaging over large fields of view. This is accomplished by projecting a two-dimensional sinusoidal pattern of light onto a sample and analyzing how the intensity of the reflected pattern changes as a function of spatial frequency. By measuring the reflectance with at least two different spatial frequencies, an inverse reflectance model can be used, in a pixel-wise manner, to rapidly produce spatially resolved maps of both the absorption and reduced scattering coefficients.^1^^,^^2^

Traditionally, SFDI is performed within the diffuse regime of light transport through the use of long wavelengths (λ≥600  nm) and low spatial frequencies ($f_{x}\leq 0.3\;{\mathrm{mm}}^{-1}$).^2^ In this regime, a majority of the photons undergo multiple scattering events prior to remission, resulting in them traveling large distances within the tissue. As such, the reflectance becomes representative of the bulk optical properties of the tissue, allowing for the forward reflectance models underlying the technique to maintain accuracy using just the absorption and reduced scattering coefficients. However, there has been increased interest in the information that can be gained by moving SFDI measurements outside the bounds of the traditional diffusion approximation.^3^^–^^6^

One of the more straightforward of these approaches is to measure the optical properties of tissue at shorter wavelengths, which can exhibit significantly increased absorption due to melanin, hemoglobin, and blood. The increased absorption prevents photons from traveling long distances, with the remaining photons composing the reflectance signal only having scattered a few times before undergoing a large-angle backscattering event and exiting the tissue. These photons, which make up what is known as the sub-diffuse reflectance, maintain a much higher dependency on the scattering phase function (SPF) of the material, which describes the probability of a photon being scattered at a particular angle. This departure from the diffusion approximation may explain the increased SFDI error that has been observed when imaging strongly absorbing tissues.^7^

In addition to the shorter wavelength regime, there has been increasing interest in leveraging the spatial frequency of the SFDI illumination to directly sample the sub-diffuse reflectance of tissue. Known as sub-diffuse SFDI, this technique leverages the fact that the applied spatial frequency of the illumination has a direct impact on the effective penetration depth. Using spatial frequencies greater than $0.5\;{\mathrm{mm}}^{-1}$, the resulting AC portion of the reflectance is only minimally scattered and thus, by definition, is sub-diffuse, regardless of the actual underlying optical properties. Due to the increased dependence on the scattering phase function, sub-diffuse SFDI has been shown to be capable of providing optical property maps of different backscattering parameters indicative of differences in tissue microstructure.^4^^–^^6^ By sweeping through a wide range of diffuse and sub-diffuse spatial frequencies, the full spatial frequency-dependent reflectance curve can be used to determine the scattering parameters at each pixel. Although this technique has been validated for a wide variety of scattering samples, little attention has been paid to the accuracy of the corresponding absorption coefficient measurements. In addition, as the focus of sub-diffuse SFDI has mainly been on measuring SPF information, it is unknown how much inaccuracy would result from using only a single sub-diffuse spatial frequency and incorrectly assuming the SPF of the sample. As such, the feasibility of using sub-diffuse SFDI to only measure the absorption and reduced scattering coefficients is unknown.

With regard to both diffuse and sub-diffuse SFDI, there is an additional factor with an impact on the overall accuracy that has yet to be fully characterized: polarization. Most regular SFDI applications make use of a pair of crossed-polarizers to reject specular reflections from impacting the demodulation and inversion processes.^2^^,^^8^ However, in addition to rejecting specularly reflected light, cross-polarization is known to preferentially reject non-diffusely scattered photons, thus blocking the collection of the sub-diffuse portion of the signal. Although this has led to many sub-diffuse SFDI systems forgoing polarizers to maintain the desired sub-diffuse signal, there has yet to be a thorough analysis on what effects different polarization states have on the accuracy of SFDI when either the underlying optical properties or the utilized spatial frequency move between being diffuse and sub-diffuse.^3^^–^^5^^,^^9^

To begin to address this lack of understanding, this work provides an experimental demonstration of the impact that using different scattering phase functions has on the accuracy of both diffuse and sub-diffuse SFDI measurements, taken both with and without cross-polarization. Using a set of 16 tissue-like optical phantoms with four levels of scattering ($\mu_{s}^{\prime }=0.35-5.5\;{\mathrm{mm}}^{-1}$) and four levels of absorption ($\mu_{a}=0.015-5.0\;{\mathrm{mm}}^{-1}$) across the visible and near-infrared wavelength range, the effects of inherently diffuse and sub-diffuse optical properties were assessed. This was multiplexed by imaging at two different spatial frequencies, $0.3\;{\mathrm{mm}}^{-1}$ and $1.0\;{\mathrm{mm}}^{-1}$, across different wavelengths, providing diffuse and sub-diffuse SFDI, and imaging with and without cross-polarization. As such, this work helps to lay the foundation for more accurate optical property mapping over a wider range of imaging parameters.

## 2 Materials and Methods

### 2.1 SFDI System and Process

All SFDI measurements within this work were performed using a single-phase, spatial frequency domain imaging system. The system contains four LED light sources, with peak wavelengths of 395 nm (M395L5, Thorlabs, Newton, New Jersey, United States), 545 nm (M565L3, Thorlabs), 625 nm (M617L3, Thorlabs), and 850 nm (M850L3, Thorlabs), that are projected onto the sample at a 40 deg angle of incidence. This oblique angle, along with the pair of crossed polarizers (LPNIRE100-B, Thorlabs), serves to minimize the effects of specular reflection. The utilized polarizers were selected as they were found to provide an extinction ratio of at least 200:1 at all four of the wavelengths of interest. Although these polarizers are known to have a degree of internal scattering, we did not observe a significant decrease in image quality due to their inclusion. Amplitude masks printed on transparency film and placed at the back focal plane of the projection arm provide the sinusoidally patterned illumination required for SFDI, with the 395 and 545 nm channels having a spatial frequency of $f_{x}=1.0\;{\mathrm{mm}}^{-1}$ and the 625 and 850 nm channels having a $f_{x}=0.3\;{\mathrm{mm}}^{-1}$ spatial frequency, at the sample. The resulting diffuse reflectance is imaged onto three wavelength-separated, spatially aligned scientific CMOS cameras (CS165MU1, Thorlabs) that capture a 62×46.5  mm area.

Demodulated DC and AC intensities are extracted from each captured frame using a 2D Fourier–domain imaging filtering process. Briefly, images were brought into frequency space using a fast 2D Fourier transform. For the $0.3\;{\mathrm{mm}}^{-1}$ images, demodulation was accomplished using the set of 2D anisotropic filters as described and recommended by Aguénounon et al.^10^ To suppress any potential harmonic frequencies that would be present due to the imperfect nature of the amplitude masks, notch filters made from inverted sine bandpass filters were included at the second and third harmonics of the detected AC frequency. For the $1.0\;{\mathrm{mm}}^{-1}$ spatial frequency images, the increased spacing of the DC and AC spatial frequencies allowed for generalized spectral notch filters to be used instead.^11^^–^^13^ To reduce any filtering artifacts, the spectral notch filters are smoothed with a 2D Gaussian filter, with a standard deviation of 1, prior to use. For all DC demodulations, the positive and negative AC frequencies are removed, along with the first pair of harmonics if still detectable. For the AC demodulation filter, the DC peak, the negative AC frequency, and both the first and second pair of harmonics, if detected, are removed.

Using these measurements, the DC and AC reflectance values at each pixel, $p_{xy}$, are obtained using a reference image of a material with known optical properties to correct for spatial variations in the projection intensity and in the modulation transfer function of the system such that

$$R_{d}(p_{xy},f_{x})=\frac{M(p_{xy},f_{x})}{M_{\mathrm{ref}}(p_{xy},f_{x})}R_{d,\mathrm{ref},\mathrm{pred}}(f_{x}),$$ (1)

where $R_{d}$ is the reflectance image of the sample; M and $M_{\mathrm{ref}}$ are the demodulated intensity images of the sample and reference, respectively; and $R_{d,\mathrm{ref},\mathrm{pred}}$ is the predicted reflectance of the reference obtained from its optical properties. From the reflectance images, maps of the absorption and reduced scattering coefficients were determined using interpolation within pre-computed look-up tables (LUTs).^14^^,^^15^ Both the predicted reflectance values and the LUTs were generated using a series of white Monte Carlo models, the construction of which is described in Sec. 2.4.

### 2.2 Phantom Materials and Fabrication

Multispectral phantoms were made using the materials and procedures outlined in our previous work.^16^ Briefly, phantoms were made using epoxy resin as the matrix material and well-characterized pigments sourced from acrylic paint as the absorbing and scattering agents. By varying the relative concentrations of the different pigments, determined through a nonnegative least-squares fit, the wavelength-dependent optical properties can be accurately controlled over a broad spectral range. The size and shape of the resulting phantoms are controlled through the use of silicone molds, which impart smooth surfaces to the phantoms free of any appreciable level of surface roughness. Although all of the previously characterized absorbing pigments were utilized in this work, only a single scatterer was chosen to simplify the expected scattering behavior of the final phantoms. Zinc oxide (ZnO, PW4) was chosen over aluminum oxide (${\mathrm{Al}}_{2}O_{3}$) and titanium oxide (${\mathrm{TiO}}_{2}$, PW6) as ${\mathrm{Al}}_{2}O_{3}$ provides too little scattering per mass fraction in epoxy to effectively reach tissue-like reduced scattering coefficients by itself and ${\mathrm{TiO}}_{2}$ has an absorption peak that encompasses the 395 nm channel on the SFDI system, making it difficult to achieve separate control over the absorption and scattering of the phantoms.

To assess the impact that the selection of scattering phase function has on SFDI accuracy, phantoms with four levels of absorption and four levels of scattering were made for a total of 16 different phantoms. These phantoms were made using a 35×35  mm mold and had an average thickness of 17 mm, which was found to be sufficiently optically thick at all four wavelengths. In addition, a small portion of each phantom material, prior to curing, was used to produce thin samples ranging in thickness from 0.3 to 1.8 mm, for the optical property characterization, described below (Sec. 2.5). With the spectral control that the multipigment phantom platform affords, the range of absorption coefficients for each of the SFDI imaging channels was independently controlled. For the 850 nm and 625 nm channels, the absorption was kept relatively low with target values falling between 0.015 and $0.1\;{\mathrm{mm}}^{-1}$ and 0.03 and $0.3\;{\mathrm{mm}}^{-1}$, respectively. For the green and blue channels, the absorption was set to relatively higher values due to the hemoglobin absorption peaks to which they correspond in tissue. For the green channel, this was represented by values ranging from 0.1 to $2.5\;{\mathrm{mm}}^{-1}$, whereas the blue channel targets were between 0.5 and $5.0\;{\mathrm{mm}}^{-1}$. As the reduced scattering coefficient at each wavelength was predominantly dependent on the concentration of zinc oxide, independent spectral control could not be achieved. This resulted in the relative scattering across the wavelength channels remaining consistent, with the target reduced scattering coefficients in the blue channel ranging from 1.25 to $5.5\;{\mathrm{mm}}^{-1}$, in the green channel from 0.8 to $3.5\;{\mathrm{mm}}^{-1}$, in the red channel from 0.55 to $3.0\;{\mathrm{mm}}^{-1}$, and in the NIR channel from 0.35 to $2.0\;{\mathrm{mm}}^{-1}$. A detailed breakdown of the target optical properties at each wavelength, for each of the 16 phantoms, can be found in Table 1.

**Table 1:** Target absorption and reduced scattering coefficients (${\mathrm{mm}}^{-1}$), and the resulting reduced albedo ($\alpha^{\prime }=\mu_{s}^{\prime }/(\mu_{a}+\mu_{s}^{\prime })$, dimensionless), for each experimental phantom.

	850 nm			625 nm			545 nm			395 nm		
	$\mu_{a}$	$\mu_{s}^{\prime }$	$\alpha^{\prime }$	$\mu_{a}$	$\mu_{s}^{\prime }$	$\alpha^{\prime }$	$\mu_{a}$	$\mu_{s}^{\prime }$	$\alpha^{\prime }$	$\mu_{a}$	$\mu_{s}^{\prime }$	$\alpha^{\prime }$
Phantom 1	0.015	0.35	0.96	0.03	0.55	0.95	0.1	0.8	0.89	0.5	1.25	0.71
Phantom 2	0.015	1.0	0.99	0.03	1.4	0.98	0.1	1.6	0.94	0.5	2.5	0.83
Phantom 3	0.015	1.5	0.99	0.03	2.2	0.99	0.1	2.6	0.96	0.5	4.0	0.89
Phantom 4	0.015	2.0	0.99	0.03	3.0	0.99	0.1	3.5	0.97	0.5	5.5	0.92
Phantom 5	0.05	0.35	0.88	0.1	0.55	0.85	0.3	0.8	0.73	1.0	1.25	0.56
Phantom 6	0.05	1.0	0.95	0.1	1.4	0.93	0.3	1.6	0.84	1.0	2.5	0.71
Phantom 7	0.05	1.5	0.97	0.1	2.2	0.96	0.3	2.6	0.90	1.0	4.0	0.80
Phantom 8	0.05	2.0	0.98	0.1	3.0	0.97	0.3	3.5	0.92	1.0	5.5	0.85
Phantom 9	0.075	0.35	0.82	0.15	0.55	0.79	1.0	0.8	0.44	2.5	1.25	0.33
Phantom 10	0.075	1.0	0.93	0.15	1.4	0.90	1.0	1.6	0.62	2.5	2.5	0.50
Phantom 11	0.075	1.5	0.95	0.15	2.2	0.94	1.0	2.6	0.72	2.5	4.0	0.62
Phantom 12	0.075	2.0	0.96	0.15	3.0	0.95	1.0	3.5	0.78	2.5	5.5	0.69
Phantom 13	0.1	0.35	0.78	0.3	0.55	0.65	2.5	0.8	0.24	5.0	1.25	0.20
Phantom 14	0.1	1.0	0.91	0.3	1.4	0.82	2.5	1.6	0.39	5.0	2.5	0.33
Phantom 15	0.1	1.5	0.94	0.3	2.2	0.88	2.5	2.6	0.51	5.0	4.0	0.44
Phantom 16	0.1	2.0	0.95	0.3	3.0	0.91	2.5	3.5	0.58	5.0	5.5	0.52

### 2.3 Scattering Phase Function Determination

The scattering phase function of zinc oxide was determined through a direct goniometric approach. As multiple scattering events result in a distortion of the measured scattering phase function when using this approach, an optically thin phantom sample consisting only of zinc oxide and epoxy resin was prepared such that the probability of multiple scattering was minimized.^17^ This was accomplished through carefully selecting both the concentration of scatterers as well as the physical thickness of the resin phantom such that any incident light experienced no more than one scattering mean free path.

Using a mass fraction of 4.165  mg/g for the ZnO acrylic paint, a reduced scattering coefficient of $∼0.334\;{\mathrm{mm}}^{-1}$ can be expected at 600 nm.^16^ Although the overall scattering anisotropy of zinc oxide has not been thoroughly characterized, it has been estimated in the literature to be between 0.8 and 0.9 using a collimated transmission and inverse adding-doubling approach.^18^^,^^19^ As such, the scattering coefficient of the phantom can be estimated to fall somewhere between 1.67 and $3.34\;{\mathrm{mm}}^{-1}$ at 600 nm. By constraining the thickness of the phantom to only 0.33 mm, the pathlength of incident light should be between 0.55 and 1.10 mean free paths, thus minimizing the influence of multiple scattering.

Using this phantom sample, goniometric measurements were made at a wavelength of 600 nm using the universal measurement accessory (UMA) of the Cary 5000 spectrophotometer (Cary 5000, Agilent, Santa Clara, California, United States). Measurements were taken using 10 deg steps from 0 deg to 60 deg and 120 deg to 170 deg and repeated for respective negative angles. The angles between 60 deg and 120 deg were omitted as the relatively large width of the phantom, compared with its thickness, served to occlude the detector from scattered light when it was near 90 deg. To ensure a precise sampling of the angle-dependent scattering, measurements were taken by placing an 8.2 mm diameter aperture in front of the detector. As the detector is located 128 mm from the sample, this resulted in the aperture subtending a solid angle of 3.2 msr. This value was used to normalize the measurements taken at each angle, resulting in the fraction of scattered light per solid angle.

To account for any inherent absorption or scattering effect of the epoxy resin itself, a pure epoxy resin sample, without any additional absorbers or scatterers, 0.37 mm thick was made. The total transmission through the clear sample was measured using the UMA with the detector at 0 deg and without the aperture in place, and each of the measurements of the ZnO sample was normalized to this measurement. In addition, the angular-dependent measurements were repeated on the clear sample with the measurements not taken at 0 deg being subtracted from their corresponding ZnO measurements, primarily serving to correct for any surface scattering effects that would artificially increase the amount of light collected at angles close to 180 deg.

**Fig. 1:**
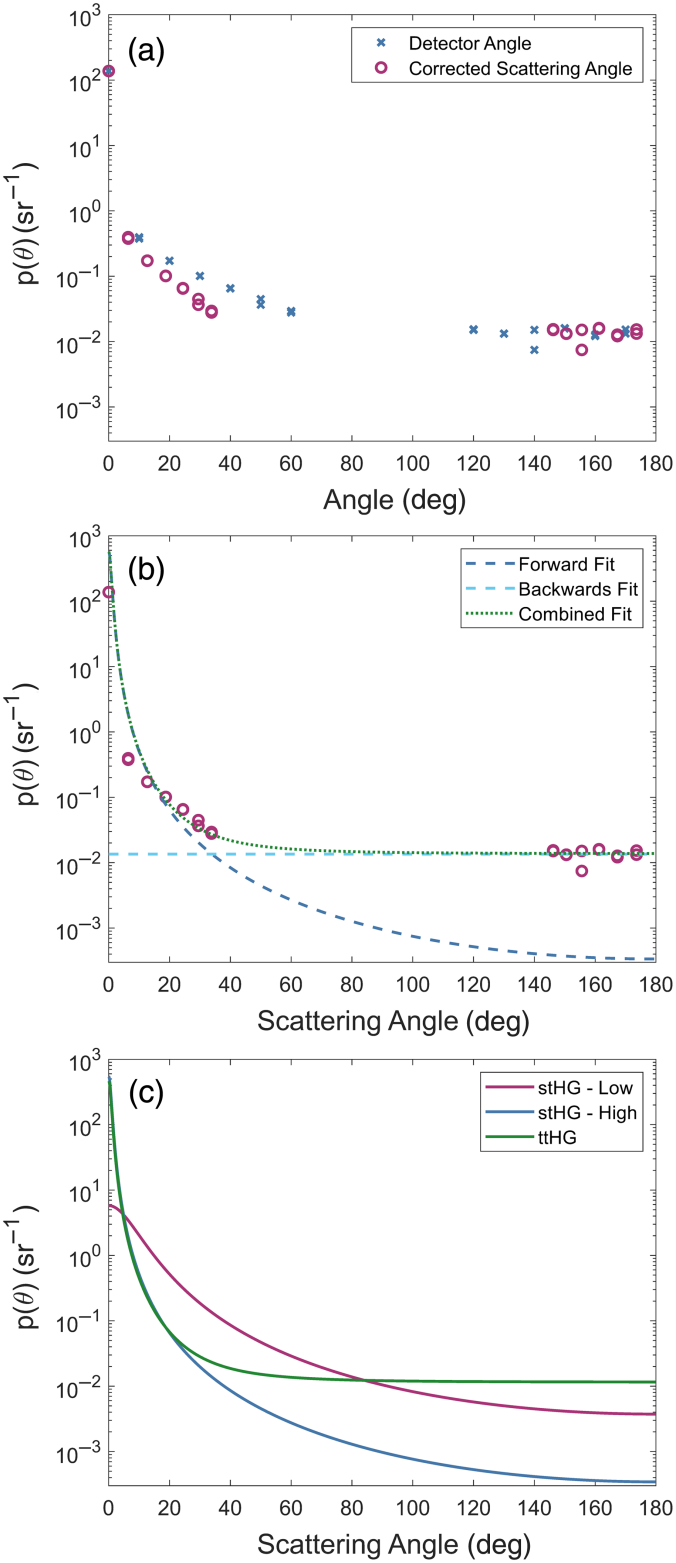
Zinc oxide scattering phase functions. (a) Comparison of the measured and corrected angle-dependent scattering behavior of zinc oxide. (b) Single-term Henyey–Greenstein phase functions are fit to the highly forward-scattering and isotropic backward-scattering behavior of zinc oxide. Their combination yields a two-term Henyey–Greenstein phase function matched to zinc oxide. (c) The three scattering phase functions used to represent the scattering behavior of zinc oxide.

The final step that was applied to the angular measurements was to convert the collection angle of the detector to the corresponding angle of the actual scattering events. As the incident light is scattered within a material with a relatively high index of refraction (n=1.556), the scattered light exiting the sample away from the normal of the sample surface undergoes a strong refraction at the air-sample interface, increasing its propagation angle away from the nearest normal. As the thickness of the sample is much smaller than the sample-to-detector distance, this change in angle is approximated through the simple Snell’s law associations:

$$\theta_{\mathrm{scat}}=\{\begin{array}{ll} \sin^{-1}(\frac{n_{\det}}{n_{\text{sample}}}\;\sin\;\theta_{\mathrm{meas}}), & 0\;\deg<\theta_{\mathrm{meas}}<90\;\deg\\ 180\;\deg-\sin^{-1}(\frac{n_{\det}}{n_{\text{sample}}}\;\sin\;\theta_{\mathrm{meas}}), & 90\;\deg<\theta_{\mathrm{meas}}<180\;\deg \end{array},$$ (2)

where $\theta_{\mathrm{meas}}$ is the angle of the detector relative to the propagation direction, $\theta_{\mathrm{scat}}$ is the angle of the scattering event, $n_{\text{sample}}$ is the refractive index of the medium in which the scattering occurs, and $n_{\det}$ is the refractive index of the medium in which the detector is located, which in our case is air.^20^^,^^21^ The effects of this angle correction can drastically change the shape of the resulting scattering phase function, as can be seen in Fig. 1(a).

To obtain the full scattering phase function of ZnO, the corrected angular-dependent measurements needed to be fit to an SPF model. Although many different models for SPF have been proposed, we chose to utilize the two-term Henyey-Greenstein phase function (ttHG) due to its relative simplicity while maintaining enough robustness to ideally match a large number of previously measured biological scattering phase functions.^22^^,^^23^ As its name implies, the ttHG is a modified version of the original Henyey-Greenstein function, hereafter referred to as a single-term Henyey-Greenstein phase function (stHG), made from the weighted sum of two stHG functions and expressed with the equation

$$p(\theta )=\frac{1}{4\pi }[\alpha \frac{1-g_{f}^{2}}{{\left(1+g_{f}^{2}-2g_{f}\;\cos\;\theta \right)}^{3/2}}+(1-\alpha )\frac{1-g_{b}^{2}}{{\left(1+g_{b}^{2}-2g_{b}\;\cos\;\theta \right)}^{3/2}}],$$ (3)

where θ is the scattering angle, $g_{f}$ is the forward-scattering anisotropy, $g_{b}$ is the backward-scattering anisotropy, and α is the fractional contribution of the forward-scattering component, which takes values between 0, indicating complete backscattering contribution, and 1, indicating complete forward-scattering contribution. The shapes of the two components are controlled by their respective anisotropies with $g_{f}$ being constrained to fall between 0 and 1 (isotropic scattering to perfect forward scattering) and $g_{b}$ being constrained between −1 and 0 (perfect backscattering to isotropic scattering). As with all SPFs, the overall anisotropy, or asymmetry parameter, of a ttHG is the average value of the cosine of the scattering angle, represented by $g_{1}$. For a ttHG, this can be quickly determined from the weighting parameter and the forward and backward anisotropies such that

$$g_{1}=\langle \cos\;\theta \rangle =\alpha g_{f}+(1-\alpha )g_{b}.$$ (4)

To fit the angular-dependent measurements of ZnO to the appropriate ttHG function, the data was first separated into the forward-scattering (θ<60  deg) and backscattering (θ>120  deg) components. Each portion was then fit to a stHG function, with an unknown scaling factor, using a nonlinear least-squares optimization. However, as the forward-scattering data spans multiple orders of magnitude, the resulting fit would overly prioritize the larger values near 0 deg. To account for this, we applied a weighting function to the fit where the weight at each angle is the maximum measurement in the section divided by the measurement at that angle. This corrects for the differences in magnitude so that each measurement is treated equally in the optimization. Once both the forward and backward stHG fits were determined, α was determined as

$$\alpha =\frac{s_{f}}{s_{f}+s_{b}},$$ (5)

where $s_{f}$ was the scaling factor on the forward stHG fit and $s_{b}$ was the scaling factor on the backward stHG fit.

Including the resulting ttHG scattering phase function, three different SPFs representing the scattering behavior of ZnO were generated. The other two chosen SPFs were stHG functions similar to what would be used more commonly in the models and simulations for SFDI. The first stHG model kept the same overall anisotropy, $g_{1}$, as the ttHG model, whereas the second stHG model utilized just the forward anisotropy, $g_{f}$, of the ttHG fit. These two SPFs are referred to throughout this work as stHG-Low and stHG-High, respectively.

### 2.4 White Monte Carlo Integration

To integrate the effects of the three scattering phase functions into the SFDI process, they need to be included in both the forward and inverse models used throughout the process. One of the most common forward models used to predict the spatial frequency-dependent reflectance behavior of a material, given its optical properties, is the white Monte Carlo (wMC) model.^24^ A wMC is a special application of the Monte Carlo method of modeling light propagation that circumvents the usual need of having to run a full simulation to obtain reflectance values for each new set of optical properties. This is accomplished by relying on a precomputed distribution of remitted photons, in both time and space, relative to a pencil beam source that is incident on a purely scattering medium. Changes to the reduced scattering coefficient can be applied to this distribution by scaling the results in both space and time such that increasing $\mu_{s}^{\prime }$ by a factor of β is accounted for by scaling both the temporal and radial distances traveled by the collected photons by a factor of 1/β. Non-zero absorption can then be applied to the distribution by utilizing the Beer-Lambert law where the distance traveled is determined from the newly scaled time of flights and the speed of light within the material.

By summing over time, the modified two-dimensional distribution can be converted into the spatially resolved diffuse reflectance, which in turn can be converted into the spatial frequency-dependent reflectance, as outlined by Cuccia et al.,^1^ using a 1D Hankel transform of order zero. However, although the wMC approach can rapidly account for changes in the density of the scattering events, the angular dependence of those events is inherent to the scattering phase function and anisotropy utilized when the original Monte Carlo simulation was run. As such, the ttHG, stHG-Low, and stHG-High scattering phase functions each required their own separate wMC models.

These wMC models were generated using the MCXLAB version of Monte Carlo eXtreme (MCX).^25^ A 300×300×500  mm volume was modeled using 2 mm-sized cubic voxels. A pencil beam source with $10^{7}\text{ photons}$ was placed incident on the volume in the center of the +z face with propagation along the −z direction, and the model was allowed to run for 300 ns of propagation with a 50 ns step size. The optical properties of the volume were homogenous and fixed with an absorption coefficient of $10^{-8}\;{\mathrm{mm}}^{-1}$, a reduced scattering coefficient of $10\;{\mathrm{mm}}^{-1}$, and a refractive index of 1.56, matched to that of epoxy resin.^16^^,^^26^ The scattering anisotropy of the volume was set as the respective $g_{1}$ for each of the three described SPFs. Although MCX, similar to a majority of available Monte Carlo software, defaults to utilizing a single-term Henyey-Greenstein phase function to probabilistically determine the photon scattering behavior, it is also able to utilize an arbitrary, user-defined phase function in the form of the corresponding inverse cumulative distribution function (CDF).

As an analytical solution to the inverse CDF of a ttHG does not exist, this function was determined numerically by first creating the ttHG function in Matlab with a dependence on the cosine of the scattering angle (e.g., p(cos(θ)) instead of p(θ)), which takes values between −1 and 1. Using this function, the CDF was determined by iteratively integrating the SPF from cos(θ)=−1 to a new upper limit using a step size of 0.0001. From this, the inverse CDF was calculated numerically by flipping the relationship between cos(θ) and the CDF before interpolating the values of cos(θ) that can be expected given a linear sampling of CDF values of Δc:Δc:(1−Δc) using a Δc value of 0.0001. The accuracy of the numerical results was validated by sampling the inverse CDF at $10^{7}$ random values between 0 and 1 and comparing the average result to the expected $g_{1}$ of the SPF before utilization in the model.^27^^,^^28^

To create the 2D photon distribution required for the white Monte Carlo, every photon remitted back through the top surface of the simulation volume was detected using a single detector centered over the top of the volume. For each detected photon, both the radial distance it had traveled from the source, calculated from its exit position, and its time-of-flight, calculated from its partial pathlength and the speed of light within the simulation volume, were recorded. Using these pairs of values, a bivariate histogram was created. The bins for each axis of the histogram were logarithmically spaced to best capture the differences in sub-diffuse scattering behavior expected between the different SPFs while maintaining the full diffuse behavior of the simulated volume and without having to work with an overly large matrix. Bin centers were set from $10^{-7}$ to 300 ns for time-of-flight and $10^{-5}$ to 150 mm for radial distance, using 750 total bins for each.

To verify that the resulting wMC models for the three SPFs provide accurate spatial frequency-dependent reflectance information, they were validated against direct Monte Carlo simulations of SFDI. Using each of the three designated SPFs, a homogenous volume was illuminated by $10^{8}\text{ photons}$ distributed as a sinusoidally patterned source using one of 10 equally spaced spatial frequencies from 0.1 to $1.0\;{\mathrm{mm}}^{-1}$. For speed, the size of the simulation volume was set to scale with the applied spatial frequency, with the length of the volume set to twice the period of the pattern, the width fixed at 1 mm, the depth set to five times the theoretical penetration depth of the volume, and the voxel size adjusted to provide 25 voxels per period. To approximate a semi-infinite domain and source, the boundary conditions on the ±x and ±y faces of the volume were set to be cyclical so that a photon that exits one face reenters from the opposite face. Each spatial frequency was simulated at three different phase shifts, 0, 2π/3, and 4π/3, and the spatially resolved diffuse reflectance was recorded for each. After scaling to the average intensity of the illumination pattern, the AC reflectance for each spatial frequency was determined using the three-phase demodulation approach, whereas the DC reflectance was approximated by averaging all three phases together.^1^ Using a fixed reduced scattering value of $5\;{\mathrm{mm}}^{-1}$, the results of this direct simulation were compared with the reflectance values predicted by the three wMC models using two different reduced scattering-to-absorption ratios, 500:1 and 5:1.

After validation, each wMC was used to create independent LUTs used as the inverse model to rapidly determine the reduced scattering and absorption coefficients from a corresponding pair of reflectance values at different spatial frequencies.^14^^,^^15^ Matching the different spatial frequencies used by the single-phase SFDI system, two sets of LUTs were made for each SPF. To account for the large range of potential reflectance values, both axes of the LUTs were sampled logarithmically, with the DC reflectance being sampled from 0.001 to 0.9 over 190 points and the AC reflectance being sampled from 0.001 to 0.7 over 170 points. Despite the nonlinear sampling, the associated 2D interpolation approach was found to still provide accurate results.

### 2.5 Experimental Phantoms

To serve as the ground truth for the SFDI comparisons, the absorption and reduced scattering coefficients of the 16 phantoms were determined at the four imaging wavelengths of 395 nm, 545 nm, 625 nm, and 850 nm. As the inverse adding-doubling approach for determining optical properties only utilizes stHG phase functions, it has shown difficulties in accurately determining the optical properties near large absorption peaks, where the material is inherently sub-diffuse, without utilizing post-processing corrections.^16^^,^^29^^–^^33^ An inverse Monte Carlo (iMC) approach was selected to determine the optical properties as the ttHG phase function for zinc oxide could be directly utilized within the forward model to more accurately account for the proportion of sub-diffuse and backscattered reflectance.

The iMC was performed directly through a nonnegative, nonlinear least-square optimization in which the outputs of a forward Monte Carlo model, with input absorption and reduced scattering coefficients that were iterated, were compared with the measured properties. To guarantee that the ground truth optical properties were as robust as possible, two different iMC approaches were utilized, with results being averaged to determine the overall ground truth for the absorption and reduced scattering coefficients for each phantom. The first iMC followed a traditional approach and mimicked the behavior of the inverse adding-doubling approach using the measured reflectance and transmittance of the thin phantom samples. The second approach took advantage of the relative robustness of iMCs and utilized the combination of diffuse reflectance from the thick phantom samples and the transflectance (the simultaneous measurement of the transmittance and reflectance) obtained from mounting the thin samples in the middle of the integrating sphere. These measurements were taken using a dual-beam spectrophotometer (Cary 5000, Agilent) with a single integrating sphere attachment (DRA-2500, Agilent) and associated small spot size kit to allow for a beam diameter of ∼1  mm on the samples. All measurements were taken across a wavelength range of 370 to 950 nm with a 1 nm step size and a 2 nm bandwidth.

To decrease the computational load of directly solving the iMCs, the forward Monte Carlo simulations were set up as minimally as possible without compromising the results. The measurements corresponding to the thin samples were modeled using $10^{5}\text{ photons}$ and a voxel size of 10  μm. While the thickness of the simulation volume was dependent on the measured thickness, the length and width were set to 2 mm with cyclic boundary conditions to have them act as semi-infinite compared with the 1 mm diameter source. The diffuse reflectance and transmittance were determined from the resulting fraction of energy escaping from the corresponding side of the sample, normalized to the incident energy. The transflectance, on the other hand, was obtained by subtracting the fraction of energy absorbed by the simulated sample from one. For the diffuse reflectance of the thick samples, the full dimensions of the blocks were modeled using a 100  μm voxel size, $10^{6}\text{ photons}$, and a uniform, 4 mm diameter source set in the center of the sample.

Despite minimizing the forward models, the iMCs were still computationally intensive enough that determining the optical properties at every wavelength was impractical despite obtaining spectral measurements for all four output parameters. Thus, only the properties expected to be seen by each of the imaging channels in the SFDI system were determined. This was accomplished by taking the weighted average of each of the measurements, using the normalized spectral profiles of each illumination LED as the weighting functions. The spectral profiles were taken using a USB spectrometer (Flame-S-VIS-NIR, Ocean Insight, Orlando, Florida, United States) and were measured at the LEDs’ respective detectors in the SFDI system to account for the spectral throughput of the system.

### 2.6 SFDI Imaging

After determining their ground truth optical properties, the 16 experimental phantoms were imaged at each of the four wavelengths of the SFDI system. To account for the large span of expected reflectance values across the phantoms, the exposure time of each image was allowed to vary such that the full dynamic range of the camera was utilized. The resulting images were normalized to their exposure time prior to demodulation to bring them back into the same relative scale. Reflectance images were obtained from the demodulated images using a large circular reference phantom with optical properties similar to phantom 11, corresponding to the third absorption group and the third reduced scattering group of the experimental phantoms (see Table 1 and Fig. 3). To determine the effect that the cross-polarization has on SFDI in the sub-diffuse regime, this process was repeated using unpolarized illumination after removing the polarizer from the projection arm. By maintaining the polarizer on the imaging arm, any effects that the internal scattering of the polarizer has on the collected images would be present in both sets of images. Thus, any differences observed between the two sets can be accurately attributed to the different polarization states.

For both polarization configurations, the sets of images were processed using the ttHG, stGH-low, and stHG-High scattering phase functions determined for ZnO, with the main differences occurring in two of the processing steps. First, the wMC corresponding to the selected SPF was used to determine the reflectance values of the reference phantom, used in Eq. 1, as well as the ground truth reflectance values for the experimental phantoms. Second, the LUTs derived from the corresponding wMCs were used for the determination of the optical property maps of each phantom.

For each of the six resulting sets of reflectance and optical property images, a 13×13  mm (300×300  pixel) region of interest in the center of the phantom was assessed by determining the mean value as well as the upper and lower quartiles of the distribution. In addition, each region of interest was compared with the expected ground truth by determining the mean absolute percent error. To compare the overall effects of SPF and polarization selection on the SFDI accuracy, the percent errors for the four wavelength channels were averaged together. This was done for the absorption coefficient, reduced scattering coefficient, DC reflectance, and AC reflectance values. Optical property maps that resulted in over 2% of the region of interest being undetermined, which occurs when the AC reflectance values are too close to, or greater than, their associated DC reflectance values, were omitted when determining the average errors.

As the final test on the impact of the SPF and polarization on the overall accuracy of SFDI, the above process was repeated using a 99% diffuse reflectance standard (USRS-99-020, LabSphere, North Sutton, New Hampshire, United States) as the reference. Both the DC and AC reflectance values of the standard were assumed to be 0.99 and invariant with regard to polarization.^34^

## 3 Results and Discussion

### 3.1 Zinc Oxide Scattering Phase Function

The measured angle-dependent scattering behavior of zinc oxide can be found in Fig. 1(a). ZnO was found to be predominantly forward scattering, as expected, while having a small contribution of undirected backscattering. Due to the high refractive index of the epoxy resin (n=1.56), the refraction–correction of the scattering angle compressed the effective sampling range by more than 25 deg on both sides of the sample. Although this reduced the effective extent of the data to which the scattering phase function could be fit, the significant change in the shape of the forward-scattering component showcases the importance of the correction.

The stHG fits to the forward and backward-scattering data can be found in Fig. 1(b) along with the resulting combined ttHG fit. After the angle correction, the forward-scattering component was found to be extremely forward-biased with an anisotropy of $g_{f}=0.983$. This is in contrast to the backward component, which was found to be roughly isotropic in shape after performing the clear sample correction, with an anisotropy of $g_{b}=0$. Using the proportionality constant of α=0.855 resulted in a ttHG SPF with an overall anisotropy of $g_{1}=0.84$, falling into the expected range of anisotropies reported in the literature for ZnO.^18^^,^^19^ The shape of the combined ttHG SPF can be compared with the associated stHG-Low and stHG-High functions, derived from the overall and forward anisotropies, in Fig. 1(c). This comparison showcases how the overall anisotropy, taken alone, is unable to fully describe the true scattering behavior of a material.

### 3.2 White Monte Carlo

**Fig. 2:**
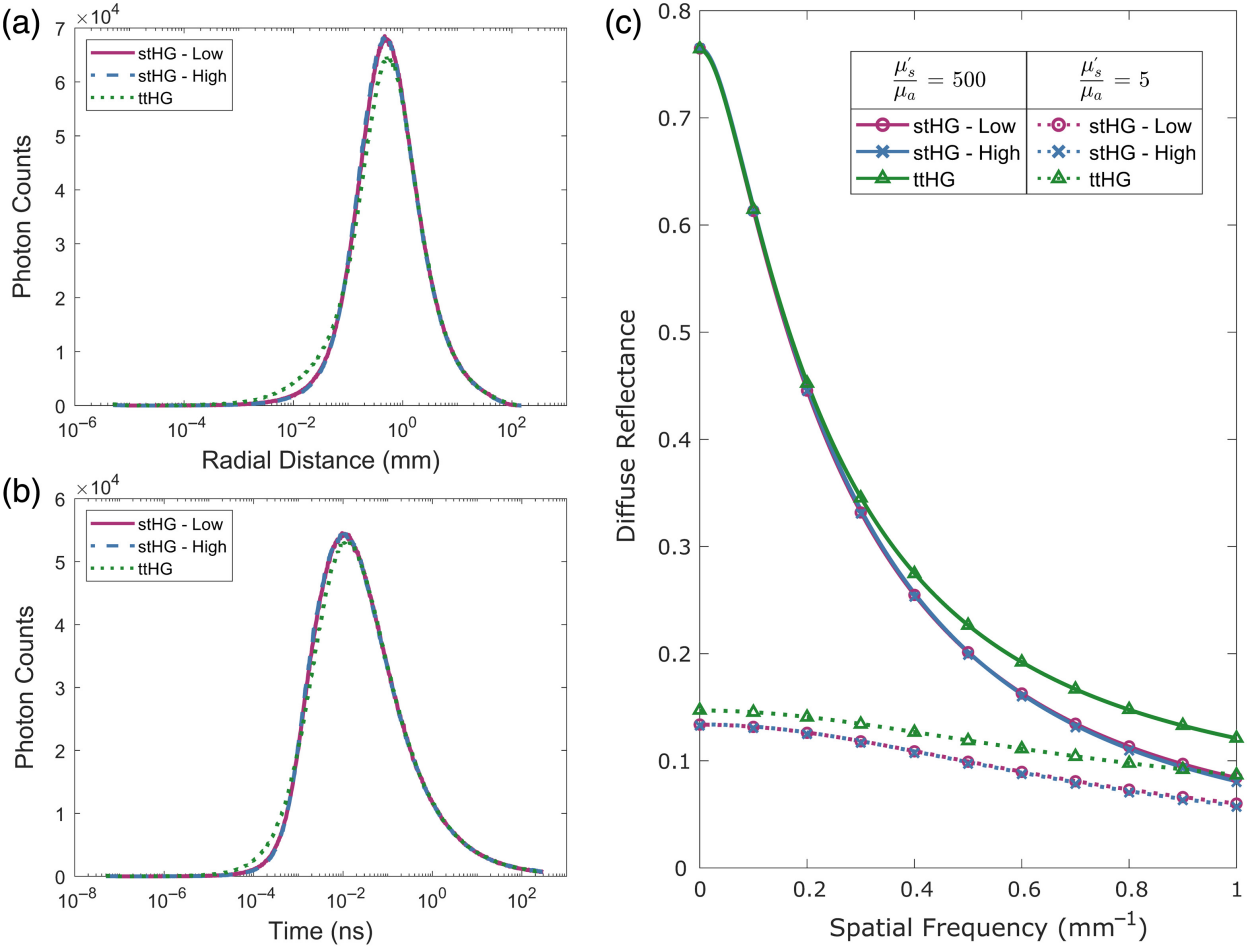
Sub-diffuse scattering white Monte Carlo. Through the use of logarithmic binning in both the radial distance (a) and time (b) axes of the white Monte Carlo photon distribution, the difference in sub-diffuse scattering behavior caused by the different scattering phase functions is resolved while maintaining the diffuse scattering behavior. (c) Comparison of the white Monte Carlo reflectance models (lines) with the direct simulation of SFDI reflectance (symbols) for both diffuse ($\mu_{s}^{\prime }/\mu_{a}=500$) and sub-diffuse ($\mu_{s}^{\prime }/\mu_{a}=5$) optical properties.

Figures 2(a) and 2(b) show the photon distributions versus radial distance and time, respectively, for the white Monte Carlo models generated using the three SPFs. From a direct comparison, it can be seen that the two different stHG phase functions produced nearly identical distributions, despite the relatively large differences in their overall anisotropies. This is most likely due to the fact that the wMC process works in the reduced scattering coefficient regime, with the original simulation being run using a fixed reduced scattering coefficient and a scattering coefficient scaled with respect to the anisotropy to achieve that value. With both stHG phase functions being predominantly forward scattering, this scaling results in distributions with negligible differences from one another.

This similarity is in contrast to the distribution that resulted from utilizing the ttHG phase function. Despite having the same overall anisotropy as stHG-Low, both the radial distance and time distributions were found to be visibly different from those of the single-term SPFs. As can be seen, these differences mainly occurred at short time and distance scales, which correspond to the more sub-diffuse and backscattering-dominated portions of the distributions.^23^ Specifically, the added backscattering behavior of the ttHG phase function resulted in an early increase in photon counts compared with the stHG, with the maximum difference occurring around 25  μm radially from the source and after a 250 fs time-of-flight. This is followed by a relative decrease in the number of remitted photons at the peak of the distributions, located near 0.5 mm and 10 ps, before realigning in the following diffuse-dominated region.

With most of the major differences mainly occurring at very small scales compared with the full simulation extent, the importance of the logarithmically spaced bins for the 2D wMC histogram must be noted. In the time-of-flight distributions, one of the major differences occurs a full six orders of magnitude below the maximum simulation time. A wMC model utilizing linear spacing would require at least $3\times 10^{6}$ bins along the time axis to preserve the sub-diffuse scattering effects of the SPFs. Coupled with the $2\times 10^{5}$ bins that would be required for an accurate linear sampling of the radial distance, the logarithmic sampling reduced the required size of the 2D distribution by a factor greater than $10^{6}$, greatly increasing its ease and speed of use.

By precisely capturing these small-scale differences, the wMC models were able to accurately predict the differences in the spatial frequency-dependent reflectance that arise due to the SPF. As can be seen in Fig. 2(c), the added backscatter component of the ttHG phase function plays a significant role in the resulting reflectance, with effects seen for both the diffuse set of optical properties, at the higher spatial frequencies, as well as for all spatial frequencies of the sub-diffuse optical property pair. In addition, it is interesting to note that, although no appreciable differences were observed in the wMC distributions between the low and high stHG phase functions, slight differences are observed in their reflectance curves at high spatial frequencies, with stHG-High having the lower relative reflectance. This difference was found to increase with increasing spatial frequency, with the maximum occurring at the largest tested spatial frequency, $f_{x}=1.0\;{\mathrm{mm}}^{-1}$, with a 3.5% difference in reflectance. With the wMC results closely matching those obtained from the direct SFDI simulations, including this slight trend between the stHG SPFs, we can conclude that this behavior is most likely an accurate representation and is thus probably due to stHG-Low having a slightly higher chance of backscattering compared with stHG-High, resulting in a marginally higher sub-diffuse reflectance.

### 3.3 Phantom Optical Properties

**Fig. 3:**
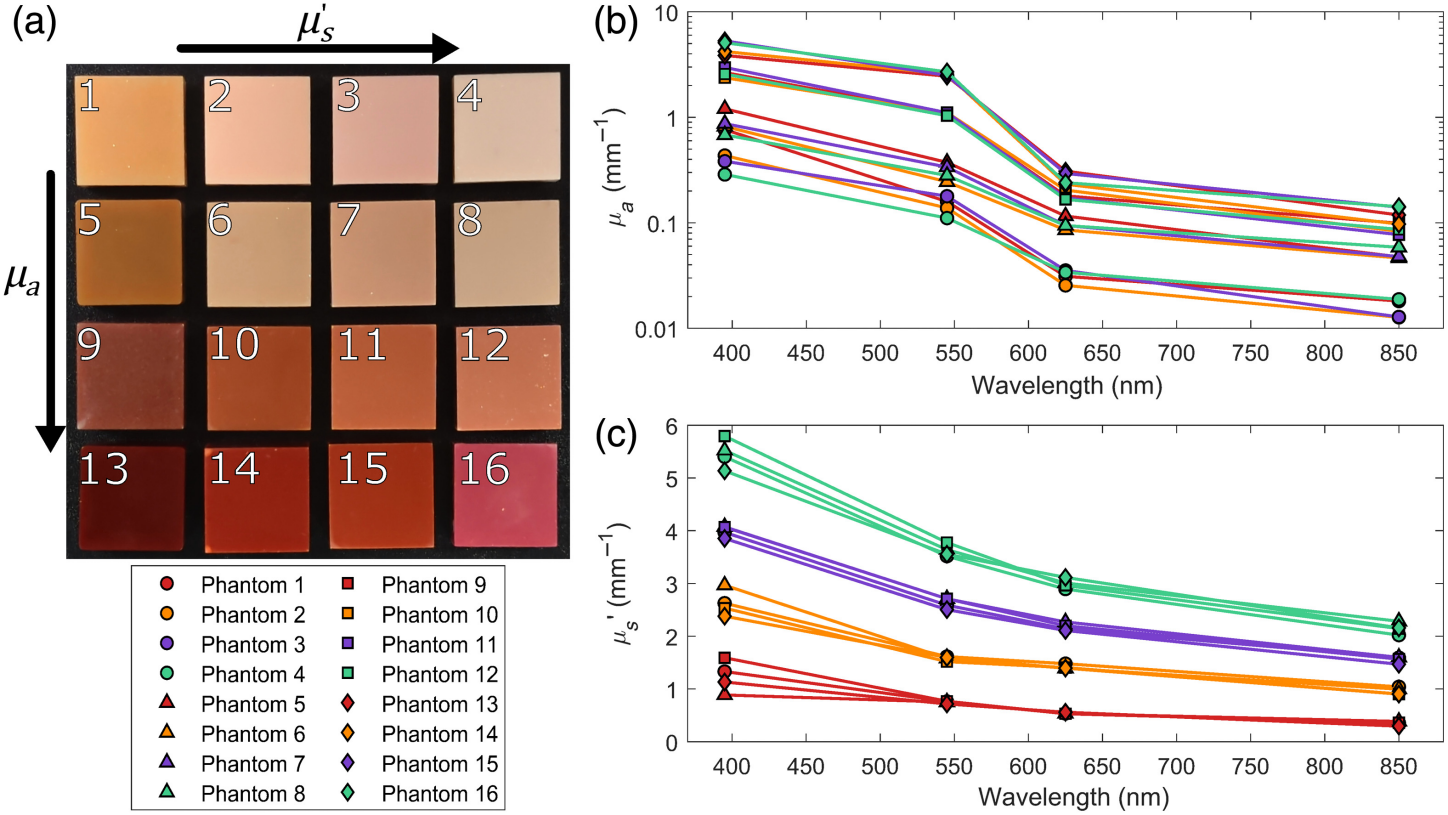
Ground truth phantom optical properties. (a) The white light image of the multipigment epoxy resin phantoms made at four levels of absorption and four levels of scattering. Each phantom is 35 mm wide. The absorption coefficient (b) and the reduced scattering coefficient (c) of each phantom were determined for each wavelength channel using an inverse Monte Carlo approach.

Figure 3 shows the 16 phantoms along with their ground truth absorption and reduced scattering coefficients determined using the described inverse Monte Carlo approach. The phantoms were found to match well with the target properties, falling distinctly into four scattering groups and four absorption groups. For the reduced scattering, the only significant variation observed was in the reduced scattering at 395 nm, with phantoms within the same group having distinctly different values, most likely due to slight miscalculations in the amount of scattering caused by the absorption-dominated pigments. On the other hand, although the measured absorption coefficients of the experimental phantoms matched well with their respective target groups, the amount of variation observed within groups is much greater, likely due to the relative strength of each of the pigments used. This caused a few phantoms to over or undershoot their target absorption coefficients at a single wavelength to such a degree that they more closely aligned with another group. Although this slightly reduces the range of optical properties being assessed, the set of phantoms still covers the wide range of optical property combinations that can be expected for a tissue at each respective wavelength; thus, they should provide a thorough assessment of the impact that both the scattering phase function and polarization have on the accuracy of SFDI.

### 3.4 Unpolarized SFDI

**Fig. 4:**
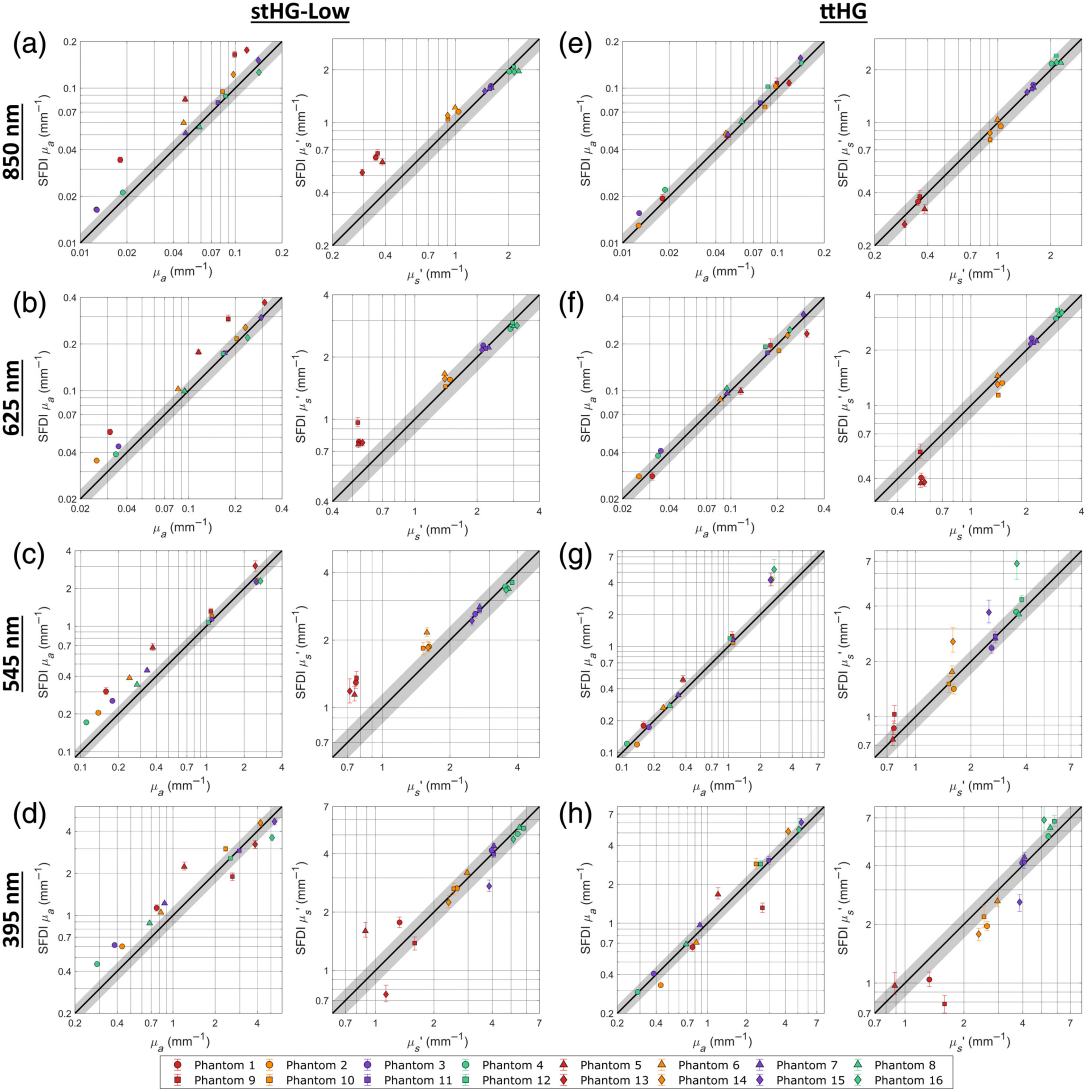
Accuracy of unpolarized SFDI using different scattering phase functions. The SFDI-derived absorption and reduced scattering coefficients are compared with the ground truth properties at the 850 nm (a), (e); 625 nm (b), (f); 545 nm (c), (g); and 395 nm (d), (h) wavelength channels when the projection arm is unpolarized. This comparison is performed when using the stHG-Low (a)–(d) and ttHG (e)–(h) scattering phase functions for the wMC model and associated LUTs. Error bars represent the upper and lower quartiles of the measured properties within the analyzed region of interest. Missing symbols represent measurements that resulted in 100% undetermined pixels.

Figures 4(a)–4(d) compare the unpolarized SFDI results of the 16 phantoms, at 850 nm, 625 nm, 545 nm, and 395 nm, respectively, to their ground truth optical properties when using the stHG-Low scattering phase function to predict the reflectance of the reference phantom and for the inversion LUTs. This is contrasted with Figs. 4(e)–4(h), which show the same comparison for the SFDI results obtained when instead using the ttHG scattering phase function. The accuracy comparison for the stHG-High SPF was found to be similar to the results when using stHG-Low. As can be seen, when SFDI imaging is performed without cross-polarization, the use of the ttHG scattering phase function results in a more accurate determination of the optical properties, which was expected due to the preservation of the sub-diffuse backscattering in the captured reflectance.^9^^,^^35^^,^^36^ The overall differences in accuracy are summarized in Table 2 with the average errors for each SPF, averaged across the four imaging channels, for the absorption coefficient; reduced scattering coefficient; DC reflectance; and AC reflectance. A detailed breakdown of the errors for each combination can be found in Table S1 in Supplementary Material. Overall, the use of the ttHG phase function resulted in at least a 60% reduction in error for both the DC and AC reflectance values, compared with using either stHG variant. This improvement is reflected in the optical property results, though reduced to only a 50% reduction in error due to the inversion process compounding on the reflectance errors.

**Table 2:** Mean absolute percentage errors of SFDI for different polarization and scattering phase function combinations using a phantom reference.

	Phantom reference							
	$\mu_{a}$ (% error)		$\mu_{s}^{\prime }$ (% error)		$R_{\mathrm{DC}}$ (% error)		$R_{\mathrm{AC}}$ (% error)	
	No input	Cross	No input	Cross	No input	Cross	No input	Cross
ttHG	**11.66**	25.26	**10.34**	26.60	**4.18**	10.42	**6.83**	21.17
stHG-Low	29.50	**12.07**	20.55	12.40	11.48	**6.48**	19.70	12.24
stHG-High	32.65	12.56	23.62	**11.95**	12.40	7.15	23.95	**11.87**

Note: bold values indicate the lowest error in each column.

When looking at Fig. 4, it can be seen that the primary effect that the choice of SPF has on the SFDI optical properties is on the reduced scattering coefficient, manifested as changes in the relative differences in the scattering groups. Visually, this appears as a change in the slope of the line made from the points of the reduced scattering accuracy plot. As the stHG phase functions do not account for the immediate backscattering behavior of ZnO, they wind up overestimating the amount of scattering required for the corresponding reflectance value, resulting in a reduction in the observed slope. However, due to the SFDI measurements being scaled to those of a reference phantom, this effect on the reduced scattering coefficient is relative to the optical properties of the reference. This results in the change in the observed scattering slope pivoting around the reference reduced scattering coefficient, which for this work is similar to the properties of phantom 11. Because of this scaling effect, the error caused from using an incorrect scattering phase function appears to be mitigated by having a reference with a reduced scattering as similar as possible to that of the sample. However, depending on the degree of mismatch between the true and utilized SPF and how sub-diffuse dominated the reflectance of the sample would be, whether due to the spatial frequency or the inherent optical properties, even small differences in the scattering can be expected to result in significant errors.

Looking at the results of the individual wavelength channels, it can be seen that, despite being relatively diffuse in both the optical properties of the phantoms as well as the utilized spatial frequency, the impact of the different SPFs is still observed at 625 and 850 nm. However, because of this diffuse nature, a greater relative distance from the reference was required for the effect to become significant, resulting in large errors only in the lowest scattering group, consisting of phantoms 1, 5, 9, and 13, when using one of the stHG variants. Though it is not directly observed due to the range of properties relative to the reference, it can be hypothesized from this behavior that samples with sufficiently higher reduced scattering coefficients than the reference would exhibit an underestimation in the SFDI-derived property when using the incorrect SPF.

At these longer wavelengths, the main errors present across the absorption coefficients are primarily caused by the observed error in the reduced scattering coefficient affecting the calculated absorption. This is caused by how the DC and AC reflectance values vary differently between the different SPFs, which can be seen in Figs. S1 and S2 in Supplementary Material. Due to the diffuse nature of the optical properties, the DC reflectance remains relatively invariant for all three SPFs. This is contrasted with the AC reflectance, which, at $f_{x}=0.3\;{\mathrm{mm}}^{-1}$, has a great enough sub-diffuse component to be impacted by the change in phase function, rotating around the reference measurement. This mismatch results in both optical properties shifting in the same direction to maintain the DC reflectance at its constant value. It can be hypothesized that using a lower spatial frequency would further mitigate the potential errors of using an incorrect SPF, given that the optical properties of the sample enable sufficiently diffuse light propagation.

In comparison with the 625 nm and 850 nm channels, both the utilized spatial frequency and the optical properties of the phantoms result in significantly more sub-diffuse light propagation at the 545 nm channel. This results in the choice of SPF having a direct impact on the DC and AC reflectance values, with both exhibiting the change in the slope of the accuracy plot, pivoting around their respective values for the reference phantom. This change is seen in the accuracy of the absorption and reduced scattering coefficients, with the pattern of the effects of a mismatched SPF being directly observed in both properties. For the reduced scattering coefficient, the observed effect is the same as seen with the previous wavelength channels, with the overall magnitude being increased due to the increase in the spatial frequency. This caused a decrease in the range at which the reference measurement mitigated the observed error, with the stHG phase functions resulting in incorrect values for both groups with lower reduced scattering coefficients than the reference. With the DC reflectance also being affected by the chosen SPFs, the overestimation of the reduced scattering coefficient no longer directly affects the paired absorption coefficients. Instead, a similar change in the relative differences in the absorption groups is observed, appearing visually as the change in slope of the accuracy plot. As observed with the reduced scattering coefficient, the reference phantom serves to mitigate the effect of the mismatched SPF, though significant errors are observed for both phantom groups with a smaller absorption coefficient than the reference. It is evident that the reference must be closely matched to both the absorption and scattering properties of the sample to fully make use of the mitigation.

Although the ttHG SPF results in a significantly lower error for the 545 nm channel, this is not without its own limitations, as can be seen in Fig. 4(g), with it resulting in large errors for both optical properties in the group of phantoms with the highest absorptions. By looking at the associated reflectance values, it can be seen that this is primarily due to the high absorption lowering the DC reflectance enough that it begins to approach that of the AC reflectance. These values being close to one another results in the inversion entering a more exponential portion of the LUTs, in which a slight variation in either reflectance value results in a significantly different pair of optical properties. It can be seen that, despite both the DC and AC reflectance values having an absolute percent error of less than 15% for the four high absorption phantoms, the determined optical properties are off by over 65%.

In addition, this proximity in the reflectance values, caused by the combination of high absorption and the ttHG phase function, results in a number of pixels with an AC reflectance greater than their associated DC reflectance. Such a combination cannot exist due to the low-pass nature of scattering samples, so the associated optical properties cannot be determined. For the 545 nm channel, all four of the high absorption phantoms had over 2% of their pixels in the defined region of interest be undetermined, with phantom 13 having completely undetermined optical properties due to its extremely low DC reflectance (see Table S1 in Supplementary Material).

This occurrence is further observed in the 395 nm channel, with the increase in absorption resulting in the proportion of pixels with undetermined optical properties increasing to over 95% for both phantoms 14 and 15. In addition, the resulting absorption to scattering ratios were so high for phantom 13 that both stHG phase functions resulted in over 50% of the pixels being undetermined in the region of interest, despite the lack of backscattering resulting in significantly reduced AC reflectance for the $1.0\;{\mathrm{mm}}^{-1}$ spatial frequency. This indicates that this issue is not unique to a particular wavelength or SPF but is likely indicative of the effective range of properties that can accurately be assessed with this current version of a single-phase SFDI system. This range could likely be further extended through the use of a more accurate demodulation technique, such as the traditional three-phase demodulation approach, to reduce any inaccuracies that may result from the 2D Fourier domain filtering, or using a reference phantom much closer in reflectance to the samples of interest, which has been shown generally to decrease measurement errors.^1^^,^^9^^,^^37^

Along with the increase in the prevalence of undeterminable optical properties, the results for the 395 nm channel appear to be less straightforward compared with the other imaging channels. For the absorption coefficient, a similar pattern as seen with the 545 nm channel is observed. The ttHG phase function was found to result in more accurate SFDI measurements, whereas the stHG variants exhibited the same change in slope around the reference, resulting an in overestimation of the lower values. Interestingly, however, all three SPFs were found to result in relatively similar amounts of error for the reduced scattering coefficient, with the stHG-Low phase function actually resulting in the overall lowest absolute percent error among the three. Comparing Figs. 2(d) and 2(h), it can be seen that the observed change in slope of the accuracy plots occurs when the ttHG phase function is used, except with the direction of the shift reversed, resulting in the low scattering values being underestimated. With none of the three SPFs resulting in an overall accurate set of optical properties, it can be hypothesized that the scattering behavior of ZnO significantly changes at shorter wavelengths, resulting in a significantly different shape to the SPF.

Overall, these results confirm the importance of including the sub-diffuse backscattering in the models used for unpolarized SFDI, with this being especially true when using higher spatial frequencies, imaging near strong absorption peaks, or otherwise not conforming to a diffuse approximation of reflectance. However, as the effects of even a low proportion of isotropic backscatter were observed to significantly affect the AC reflectance, and thus the resulting optical properties, for samples both with diffuse optical properties and imaged at a spatial frequency often assumed to be diffuse, the use of the proper SPF appears to be of importance for all varieties of unpolarized SFDI.^3^^–^^5^^,^^9^^,^^37^

### 3.5 Cross-Polarized SFDI

**Fig. 5:**
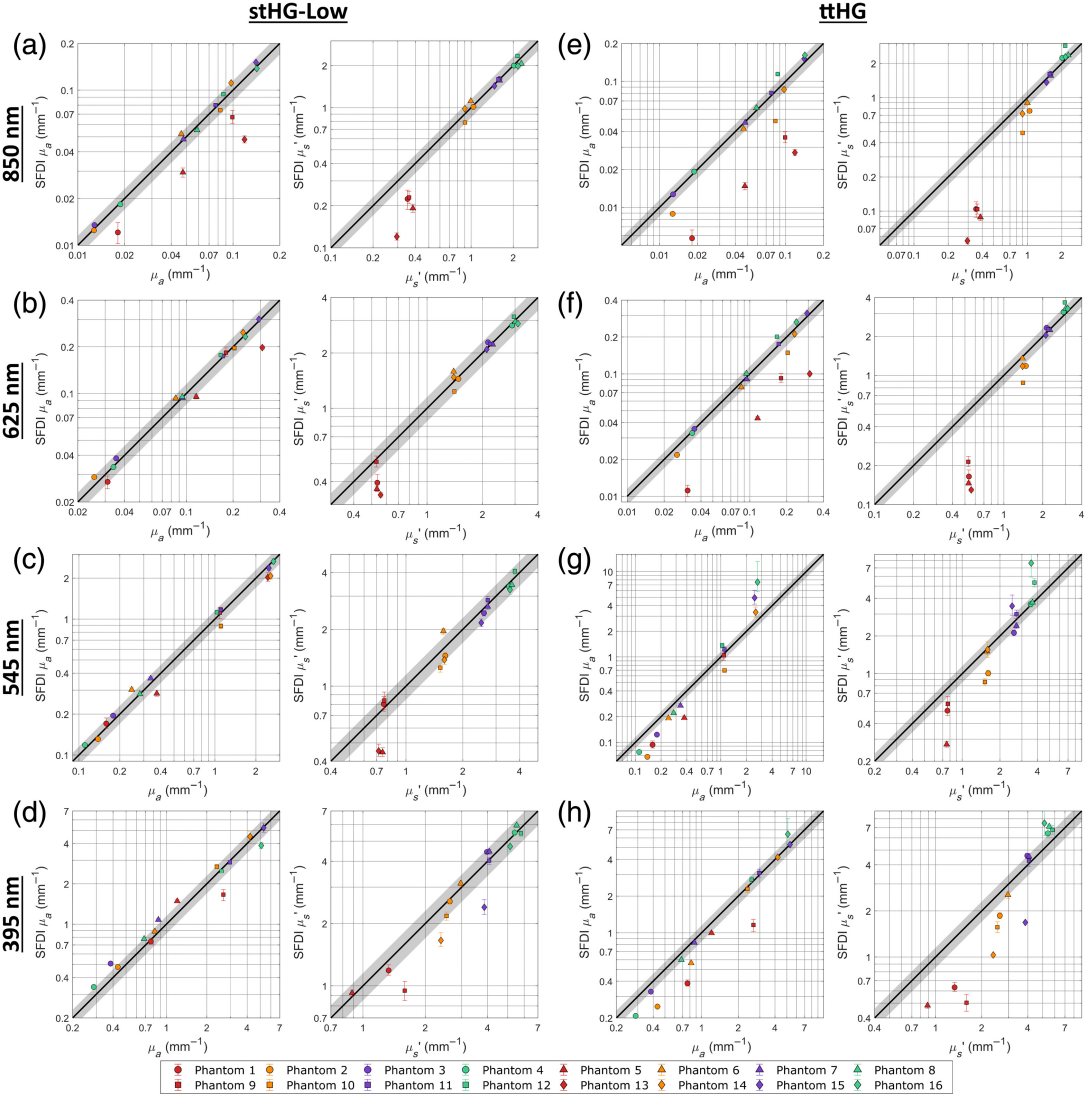
Accuracy of cross-polarized SFDI using different scattering phase functions. The SFDI-derived absorption and reduced scattering coefficients are compared with the ground truth properties at the 850 nm (a), (e); 625 nm (b), (f); 545 nm (c), (g); and 395 nm (d), (h) wavelength channels when the projection arm and imaging arm are cross-polarized. This comparison is performed when using the stHG-Low (a)–(d) and ttHG (e)–(h) scattering phase functions for the wMC model and associated LUTs. Error bars represent the upper and lower quartiles of the measured properties within the analyzed region of interest. Missing symbols represent measurements that resulted in 100% undetermined pixels.

In contrast to the previous section, Figs. 5(a)–5(d) compare the accuracy of cross-polarized SFDI at the 850, 625, 545, and 395 nm channels, respectively, when using the stHG-Low wMC and associated LUTs, and Figs. 5(e)–5(h) compare the accuracy of the SFDI optical properties when using the ttHG-associated models. The results for the stHG-High SPF were found to be similar to those of stHG-Low. Overall, it was found that, when the projection arm and imaging arm of the SFDI system are sufficiently cross-polarized, the use of the stHG scattering phase functions resulted in a more accurate determination of the optical properties as opposed to the ttHG SPF, which more accurately describes the scattering behavior of the ZnO. This is likely caused by the cross-polarizers blocking the immediately backscattered photons that predominately make up the sub-diffuse reflectance. Because these photons undergo far fewer scattering events than the diffuse component of the reflectance, they undergo a lesser degree of depolarization.^35^^,^^38^ With the suppression of the backscattering, the captured scattering behavior would only include the forward-scattering component and thus would be more accurately represented by the stHG variants, as can be seen in Figs. S3 and S4 in Supplementary Material.

The results of the different SPFs are summarized in Table 2 for both the optical property and reflectance pairs, and the error for each phantom can be found in detail in Table S2 in Supplementary Material. Despite having significant differences in their overall scattering anisotropy, no significant differences were observed between the results obtained from using the stHG-Low and stHG-High phase functions, with stHG-Low being marginally more accurate for the absorption coefficient and the DC reflectance and stHG-High being more accurate for the reduced scattering coefficient and AC reflectance values. This split could indicate that neither of these scattering phase functions truly represents the cross-polarized scattering behavior of ZnO and instead are just more accurate than the ttHG alternative. Alternatively, the SFDI results from using only a single pair of spatial frequencies may be too insensitive to the relative differences in highly forward-scattering behavior, resulting in a wide range of anisotropies yielding acceptably accurate optical properties. This would allow for cross-polarized SFDI to be used on samples with unknown scattering behavior and still yield acceptably accurate optical properties.

Compared with the unpolarized SFDI results, the effects of using an inaccurate SPF are broadly the same, but with the results reversed. With the ttHG phase function still including the now suppressed sub-diffuse component, its use results in a general underestimation of scattering values below that of the reference and an overestimation for those above. Although the reference phantom still serves to anchor the relative changes, mitigating the total amount of error observed, the degree of inaccuracy caused from using the ttHG SPF when cross-polarized is high enough that practically all of the phantoms not within the same scattering group as the reference are inaccurately determined. This further illustrates the limitations of attempting to use the reference measurement to mitigate any effects caused by using an inaccurate SPF.

Looking at the 850 nm and 625 nm wavelength channels separately, it can be observed that, although the stHG phase functions produce more accurate results than the ttHG, they still exhibit an underestimation in the lowest scattering group. Following the observed trend in how the slope of the accuracy plot changes with respect to how the utilized SPF is inaccurate, it can be hypothesized that an SPF even more forward-scattering dominated than the stHG high-phase function, which has an $g_{1}=0.985$, may best represent the cross-polarized scattering behavior of ZnO. In addition, this observed inaccuracy is more prominent at 850 nm, indicating that the scattering behavior of ZnO may become more forward-dominated at longer wavelengths.

For the 545 and 395 nm channels, despite both the optical properties of the phantoms and the utilized spatial frequency being inherently sub-diffuse dominated, the cross-polarization was effective enough to sufficiently remove the backscatter component of the reflectance, resulting in the stHG variants being overall more accurate. In addition, the ambiguity observed in the unpolarized SFDI results at 395 nm is not repeated here. It can be concluded that whatever confounded the results was eliminated after cross-polarization. This suggests that the issue was most likely due to a shift in the backscattering portion of the scattering phase function and not some other aspect of scattering behavior. However, with the cross-polarization suppressing the sub-diffuse photons, the high absorption-to-scattering ratios of the phantoms caused even lower reflectance values than previously, resulting in a number of phantoms still having high proportions of undermined optical properties (see Table S2 in Supplementary Material). However, as before, the stHG phase functions resulted in a lower proportion of undetermined optical properties compared with the ttHG, with only phantom 13 being significantly impacted at 395 nm. This means that, with the current SFDI system, accurate optical property maps can be obtained for a wider range of properties using cross-polarization to discard the sub-diffuse reflectance components.

Overall, there are two main conclusions that can be drawn from the results of the cross-polarized SFDI. First, the decision that many groups have made to not utilize crossed polarizers when using SFDI to directly probe the sub-diffuse scattering properties of an unknown sample has been further confirmed. Maintaining cross-polarization would suppress backscattering, and differences in polarization are likely the reason that different studies have resulted in disparate sub-diffuse properties for similar samples.^3^^–^^6^ Second, when the SPF for either the reference or sample is unknown, using a cross-polarized configuration would suppress any effects of backscattering, allowing basic forward-scattering approximations to provide accurate results over a wider range of optical properties and wavelengths than previously thought, though further validation using a broader variety of samples is required to confirm this.

### 3.6 SFDI with Reflectance Standard Reference

As a final assessment on the impact that the choice of SPF has on SFDI accuracy, the previously performed tests were repeated using a 99% Spectralon diffuse reflectance standard as the reference. As Spectralon has been shown to be a highly depolarizing material, it can be assumed that its relative reflectance does not vary between the unpolarized and cross-polarized SFDI configurations.^34^ Thus, given the assumption that both the DC and AC reflectance values are fixed at 0.99, the standard serves as a reference measurement unbiased by either the polarization state or the utilized SPFs.

**Fig. 6:**
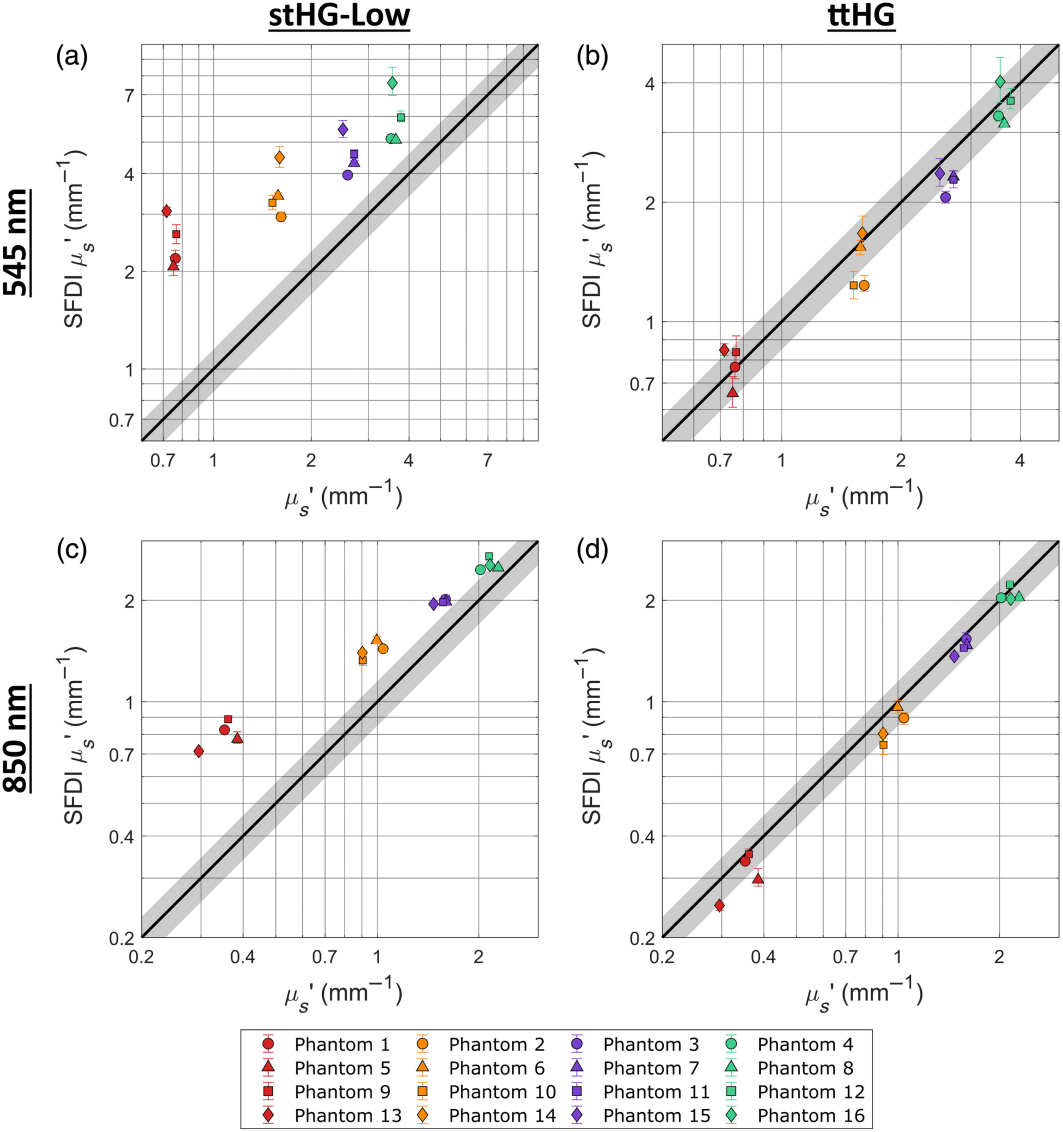
Accuracy of unpolarized SFDI when using a reflectance standard reference. The reduced scattering coefficients obtained from unpolarized SFDI are compared with the ground truth properties at the 545 nm (a), (b) and 850 nm (c), (d) channels when using a 99% reflectance standard for the normalizing reference measurement. This comparison is performed using both the stHG-Low (a), (c) and the ttHG (b), (d) scattering phase functions. Error bars represent the upper and lower quartiles of the measured properties within the analyzed region of interest.

**Fig. 7:**
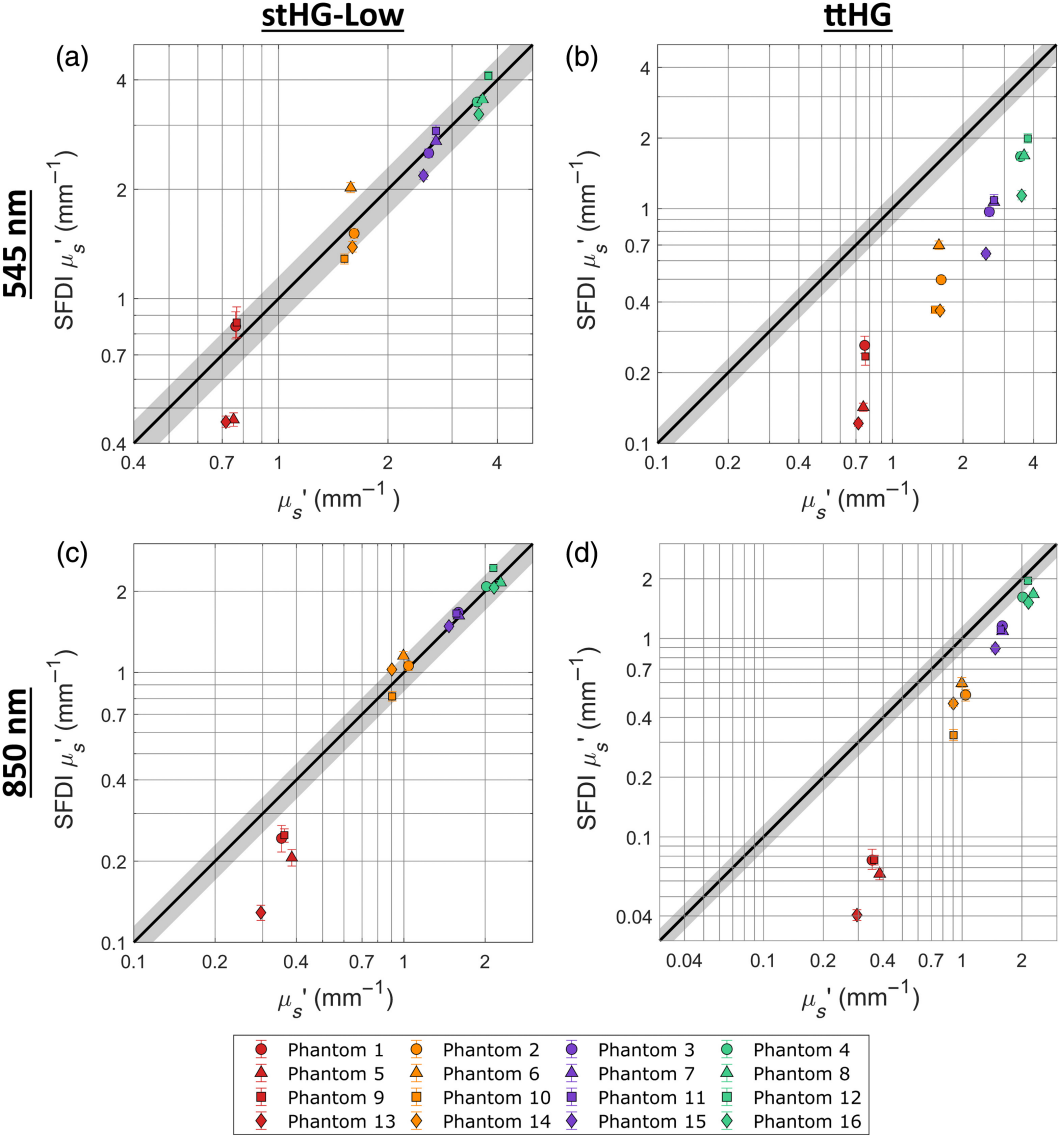
Accuracy of cross-polarized SFDI when using a reflectance standard reference. The reduced scattering coefficients obtained from cross-polarized SFDI are compared with the ground truth properties at the 545 nm (a), (b) and 850 nm (c), (d) channels when using a 99% reflectance standard for the normalizing reference measurement. This comparison is performed using both the stHG-Low (a), (c) and the ttHG (b), (d) scattering phase functions. Error bars represent the upper and lower quartiles of the measured properties within the analyzed region of interest.

Figure 6 compares the accuracy of the determined reduced scattering coefficients for unpolarized SFDI, at the 545 nm and 850 nm channels, using both the stHG-Low and ttHG scattering phase functions, and Fig. 7 does the same for cross-polarized SFDI. From these two figures, it can be seen that the same combinations of SPF and polarization as before resulted in accurate optical property determination, with the ttHG SPF being more accurate for unpolarized SFDI and the stHG SPFs being more accurate when cross-polarized (see Table 3). Interestingly, due to removing the mitigation effect caused by using a diffusely scattering phantom as the reference, the errors resulting from using a mismatched SPF were found to be significantly higher than observed previously. Using the stHG phase functions for unpolarized SFDI resulted in an overestimation of the reduced scattering of all 16 phantoms, whereas using the ttHG phase function for cross-polarized SFDI resulted in underestimating all of the scattering values. This lack of error mitigation is likely why the use of such reflectance standards in SFDI has been relatively minimal.

**Table 3:** Mean absolute percentage errors of SFDI for different polarization and scattering phase function combinations using a reflectance standard reference.

	Standard reference							
	$\mu_{a}$ (% error)		$\mu_{s}^{\prime }$ (% error)		$R_{\mathrm{DC}}$ (% error)		$R_{\mathrm{AC}}$ (% error)	
	No input	Cross	No input	Cross	No input	Cross	No input	Cross
ttHG	**10.78**	48.14	**9.90**	47.98	**3.16**	9.68	**6.96**	38.81
stHG-Low	48.35	12.32	69.50	13.42	11.90	6.00	68.11	**13.35**
stHG-High	55.90	**10.59**	77.98	**13.17**	12.49	**5.98**	82.61	14.63

Note: bold values indicate the lowest error in each column.

In addition, this consistency in behavior was found to not hold for the 395 nm channel, with none of the tested SPFs providing accurate optical properties for either polarization configuration. Due to this overall inaccuracy, the 395 nm results were left out of the wavelength-averaged errors presented in Table 3. As the stHG phase functions resulted in accurate optical properties for cross-polarized SFDI when using a phantom reference, this deviation away from the previously observed patterns must be due to something involving the standard reference. The first possibility is that the AC reflectance of the standard is no longer close to the assumed value of 0.99 due to a change in its scattering behavior at shorter wavelengths. As there is not yet a thorough understanding of the scattering phase function of Spectralon, its theoretical spatial frequency-dependent reflectance cannot be accurately determined. The other potential possibility is that the 395 nm light is inducing a relatively strong autofluorescence signal in the epoxy resin phantoms, which would be confounded with the reflectance due to the 395 and 545 nm channels sharing a detector. While this autofluorescence would be mostly normalized out when using a reference with a similar autofluorescence signal, the use of a reference without autofluorescence, such as the reflectance standard, would result in an overestimation in the reflectance values, such as what was observed. Further tests would be required to determine which, if any, of these reasons is the cause for the observed inaccuracies. Overall, however, the accuracy observed for the other three wavelength channels confirms the previously observed interactions between polarization and scattering phase function for SFDI. In addition, it showcases that it is possible to use a reflectance standard as the SFDI reference measurement to produce accurate optical property maps for a wide range of wavelengths, spatial frequencies, and optical properties, given the proper SPF is used for the LUTs.

## 4 Conclusion

This work has provided the first investigation into the effects that varying the modeled scattering phase function has on the accuracy of both diffuse and sub-diffuse SFDI under different polarization states. It was shown that, despite zinc oxide having a relatively small proportion of backscattering, the full ttHG phase function provided a much more accurate model of the reflectance for all phantoms and at all three spatial frequencies for unpolarized SFDI, resulting in overall more accurate optical properties. On the other hand, it was shown that sufficient cross-polarization was capable of removing the sub-diffuse component of reflectance, even when both the optical properties and SFDI spatial frequency were inherently sub-diffuse. This resulted in all cross-polarized SFDI being more accurate when modeled with either of the forward-scattering stHG phase functions, indicating that the complications provided by sub-diffuse scattering can be safely ignored under the right parameters. Finally, although the use of a reference phantom was seen to mitigate the effects of inaccurately matching the SPF to the polarization state, correct pairings of polarization and SPF were shown to produce accurate optical properties, even when normalized to a diffuse reflectance standard, removing the need to use relatively subjective phantom references. Overall, this work has laid the groundwork for a more thorough understanding of the processes involved in SFDI, which should enable the continual improvement of the highly impactful imaging modality.

## Supplementary Material



## Data Availability

All relevant code, data, and materials are available from the authors upon request from the corresponding author.
