# Supplementary material for: Impact of scattering phase function and polarization on the accuracy of diffuse and sub-diffuse spatial frequency domain imaging
Source: J Biomed Opt. 2024 Sep 6;29(9):095001. doi: 10.1117/1.JBO.29.9.095001 (PMC11379407; doi:10.1117/1.JBO.29.9.095001)
Supplement: Supplementary file 1 [file JBO_029_095001_SD001.pdf]

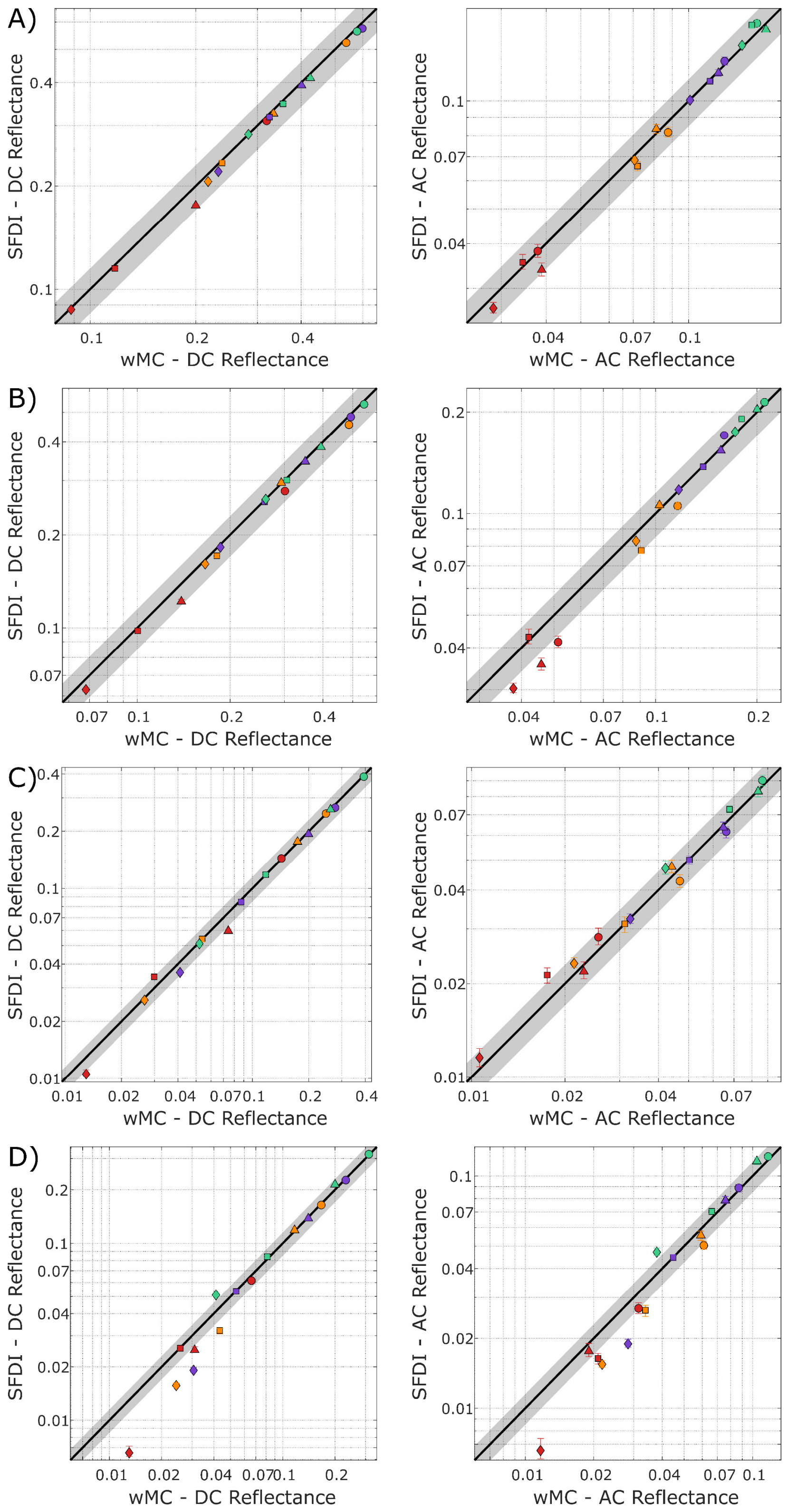


**Figure S1. Reflectance accuracy of unpolarized SFDI using the ttHG SPF.** The SFDI-derived DC and AC reflectance are compared to the wMC reflectance values at the 850 nm (A), 625 nm (B), 545 nm (C), and 395 nm (D) wavelength channels.

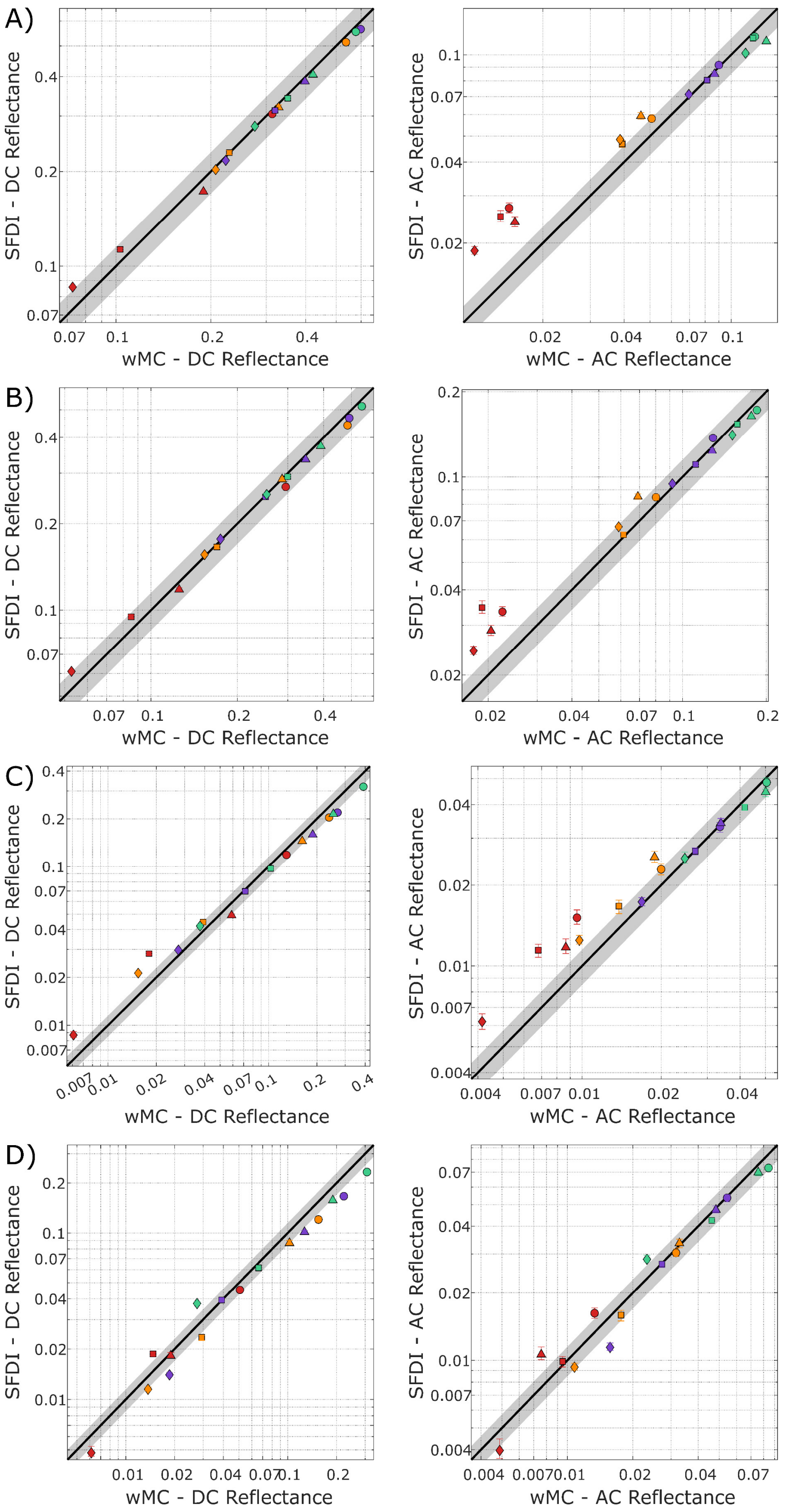


**Figure S2. Reflectance accuracy of unpolarized SFDI using the stHG-Low SPF.** The SFDI derived DC and AC reflectance are compared to the wMC reflectance values at the 850 nm (A), 625 nm (B), 545 nm (C), and 395 nm (D) wavelength channels.

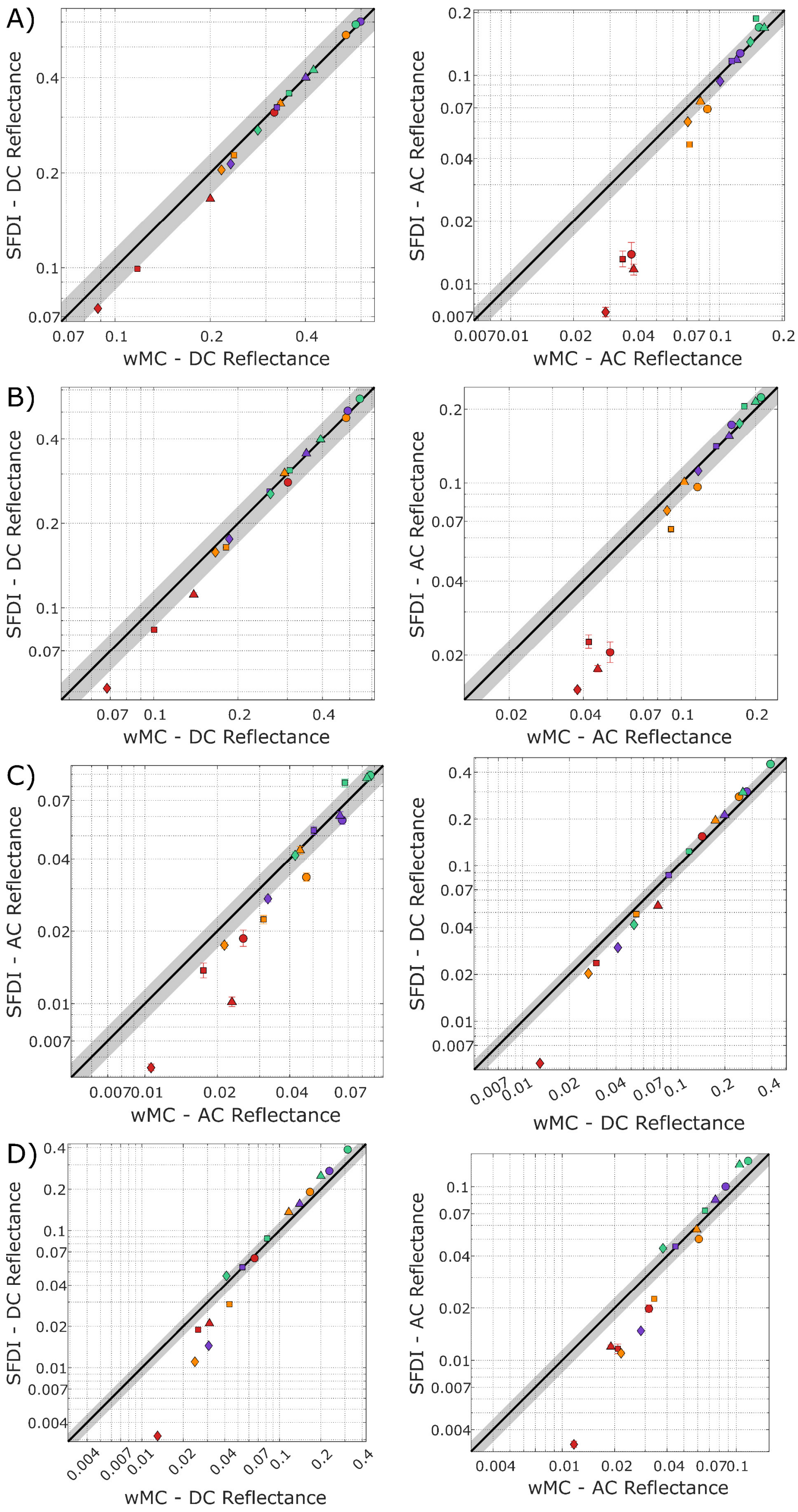


**Figure S3. Reflectance accuracy of cross-polarized SFDI using the ttHG SPF**. The SFDI-derived DC and AC reflectance are compared to the wMC reflectance values at the 850 nm (A), 625 nm (B), 545 nm (C), and 395 nm (D) wavelength channels.

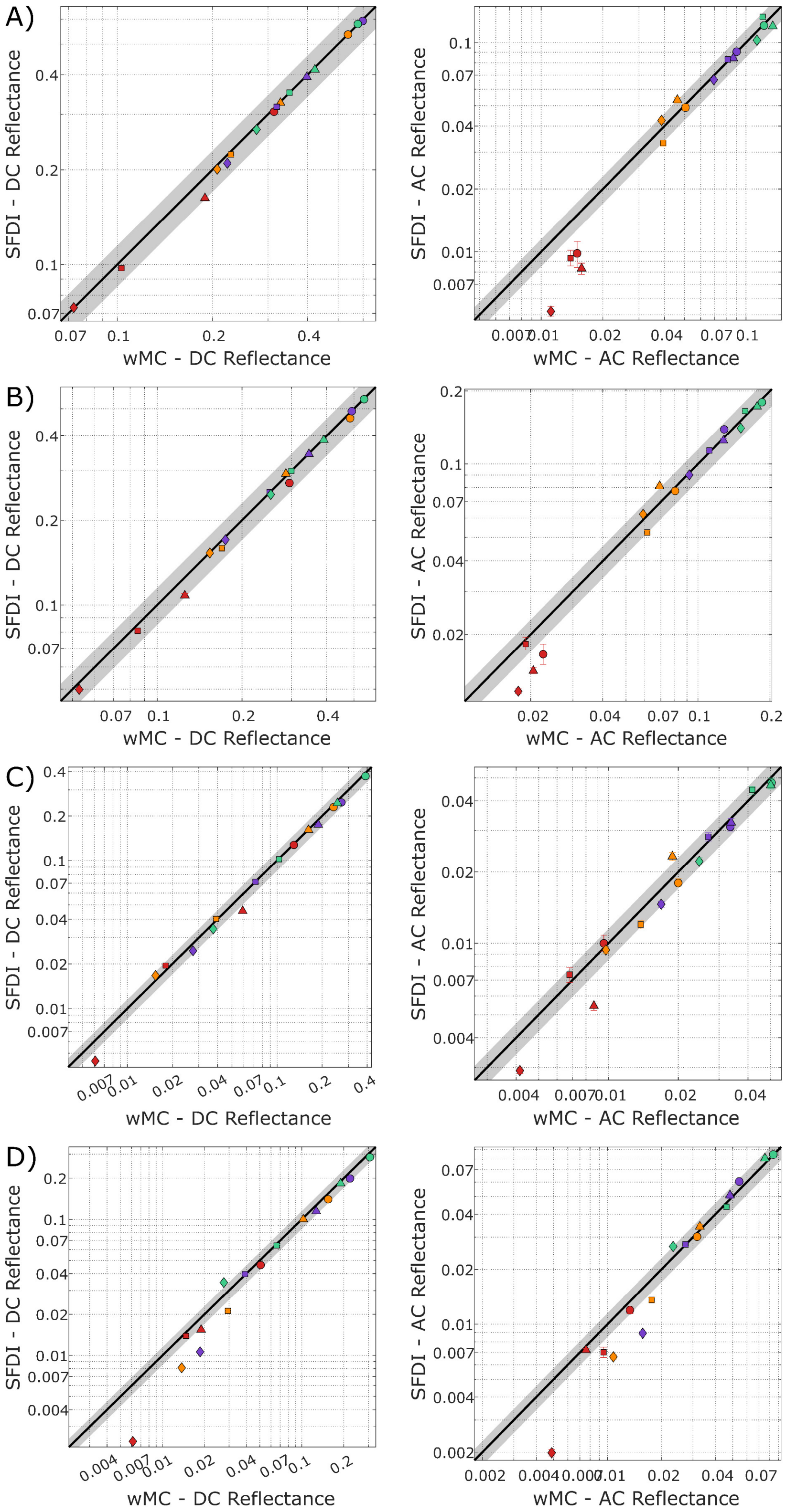


**Figure S4. Reflectance accuracy of cross-polarized SFDI using the stHG-Low SPF.** The SFDI-derived DC and AC reflectance are compared to the wMC reflectance values at the 850 nm (A), 625 nm (B), 545 nm (C), and 395 nm (D) wavelength channels.

# Table S1. Absolute percentage errors of SFDI using a phantom reference and no input polarization for different scattering phase functions.

**SPF = ttHG**

| | 395 nm | | | | | 545 nm | | | | | 625 nm | | | | | 850 nm | | | | |
|---|---|---|---|---|---|---|---|---|---|---|---|---|---|---|---|---|---|---|---|---|
| | $\lvert\mu_a$ Error$\rvert$ (%) | $\lvert\mu'_s$ Error$\rvert$ (%) | $\lvert R_{dc}$ Error$\rvert$ (%) | $\lvert R_{ac}$ Error$\rvert$ (%) | Percent NaN (%) | $\lvert\mu_a$ Error$\rvert$ (%) | $\lvert\mu'_s$ Error$\rvert$ (%) | $\lvert R_{dc}$ Error$\rvert$ (%) | $\lvert R_{ac}$ Error$\rvert$ (%) | Percent NaN (%) | $\lvert\mu_a$ Error$\rvert$ (%) | $\lvert\mu'_s$ Error$\rvert$ (%) | $\lvert R_{dc}$ Error$\rvert$ (%) | $\lvert R_{ac}$ Error$\rvert$ (%) | Percent NaN (%) | $\lvert\mu_a$ Error$\rvert$ (%) | $\lvert\mu'_s$ Error$\rvert$ (%) | $\lvert R_{dc}$ Error$\rvert$ (%) | $\lvert R_{ac}$ Error$\rvert$ (%) | Percent NaN (%) |
| **1** | 12.89 | 19.44 | 6.53 | 13.60 | 0.00 | 16.63 | 17.66 | 0.47 | 12.28 | 0.00 | 9.18 | 23.62 | 8.17 | 18.38 | 0.00 | 8.12 | 2.22 | 2.56 | 1.36 | 0.00 |
| **2** | 23.24 | 24.89 | 1.42 | 17.12 | 0.00 | 12.18 | 11.55 | 0.37 | 8.50 | 0.00 | 9.60 | 10.18 | 6.25 | 9.17 | 0.00 | 2.24 | 7.70 | 2.82 | 6.30 | 0.00 |
| **3** | 6.34 | 3.67 | 1.43 | 2.21 | 0.00 | 2.55 | 9.13 | 3.58 | 7.01 | 0.00 | 15.95 | 8.45 | 2.04 | 6.83 | 0.00 | 21.93 | 3.36 | 3.86 | 2.61 | 0.00 |
| **4** | 2.32 | 4.54 | 1.04 | 3.72 | 0.00 | 10.25 | 5.97 | 1.54 | 4.45 | 0.00 | 12.51 | 2.85 | 2.39 | 1.88 | 0.00 | 17.57 | 7.43 | 2.20 | 6.30 | 0.00 |
| **5** | 44.40 | 16.52 | 18.55 | 5.24 | 0.00 | 34.46 | 4.38 | 19.20 | 2.49 | 0.00 | 13.42 | 28.08 | 12.32 | 21.35 | 0.00 | 5.26 | 14.62 | 12.16 | 12.16 | 0.00 |
| **6** | 13.63 | 11.18 | 1.99 | 5.94 | 0.00 | 8.76 | 11.89 | 1.74 | 7.98 | 0.00 | 2.88 | 3.95 | 0.55 | 3.09 | 0.00 | 10.11 | 4.50 | 2.32 | 3.06 | 0.00 |
| **7** | 10.78 | 8.43 | 1.49 | 4.01 | 0.00 | 4.51 | 0.73 | 3.12 | 1.18 | 0.00 | 1.84 | 1.58 | 1.43 | 1.64 | 0.00 | 4.99 | 0.94 | 2.21 | 1.12 | 0.00 |
| **8** | 0.87 | 12.94 | 6.92 | 10.84 | 0.00 | 1.33 | 1.87 | 0.30 | 1.44 | 0.00 | 10.07 | 3.50 | 2.36 | 1.65 | 0.00 | 3.92 | 3.86 | 2.80 | 3.79 | 0.00 |
| **9** | 49.94 | 50.20 | 0.73 | 21.47 | 0.00 | 16.79 | 36.32 | 14.93 | 21.85 | 0.00 | 11.42 | 7.88 | 2.45 | 3.28 | 0.00 | 10.82 | 7.36 | 2.29 | 4.00 | 0.00 |
| **10** | 26.53 | 9.07 | 26.11 | 21.38 | 0.43 | 0.87 | 1.29 | 0.05 | 0.18 | 0.00 | 10.99 | 18.72 | 5.60 | 14.08 | 0.00 | 8.33 | 11.21 | 1.78 | 8.29 | 0.00 |
| **11** | 5.96 | 5.49 | 0.50 | 0.30 | 0.05 | 5.40 | 2.01 | 2.59 | 0.36 | 0.00 | 2.14 | 0.27 | 1.23 | 0.69 | 0.00 | 4.45 | 0.71 | 2.29 | 1.04 | 0.00 |
| **12** | 13.27 | 18.42 | 3.52 | 7.03 | 0.00 | 15.63 | 16.12 | 0.28 | 8.06 | 0.00 | 15.76 | 12.20 | 1.44 | 6.49 | 0.00 | 18.31 | 12.71 | 2.04 | 8.95 | 0.00 |
| **13** | | | 47.27 | 40.33 | 100.00 | | | 16.64 | 11.58 | 100.00 | 23.53 | 30.55 | 7.70 | 19.52 | 0.00 | 8.07 | 9.44 | 1.18 | 6.68 | 0.00 |
| **14** | 21.80 | 25.00 | 34.84 | 28.08 | 96.43 | 77.30 | 73.03 | 1.89 | 10.11 | 8.44 | 1.85 | 6.49 | 3.09 | 5.15 | 0.00 | 6.07 | 2.66 | 4.90 | 2.82 | 0.00 |
| **15** | 16.75 | 29.00 | 36.13 | 31.61 | 95.51 | 91.74 | 67.61 | 11.14 | 0.92 | 2.97 | 5.93 | 2.68 | 1.87 | 0.44 | 0.00 | 11.31 | 1.70 | 4.98 | 0.34 | 0.00 |
| **16** | 19.91 | 54.82 | 24.93 | 26.07 | 3.35 | 133.80 | 130.11 | 1.58 | 13.11 | 7.69 | 3.07 | 3.10 | 0.06 | 1.61 | 0.00 | 2.12 | 2.17 | 0.05 | 1.49 | 0.00 |
| **Average** | **17.91** | **19.57** | **13.34** | **14.93** | | **28.81** | **25.98** | **4.96** | **6.97** | | **9.38** | **10.26** | **3.69** | **7.20** | | **8.98** | **5.79** | **3.15** | **4.39** | |
| **Average subset** | 17.51 | 15.40 | 5.85 | 9.40 | | 10.78 | 9.91 | 4.01 | 6.31 | | 9.38 | 10.26 | 3.69 | 7.20 | | 8.98 | 5.79 | 3.15 | 4.39 | |

**SPF = stHG-Low**

| | 395 nm | | | | | 545 nm | | | | | 625 nm | | | | | 850 nm | | | | |
|---|---|---|---|---|---|---|---|---|---|---|---|---|---|---|---|---|---|---|---|---|
| | $\lvert\mu_a$ Error$\rvert$ (%) | $\lvert\mu'_s$ Error$\rvert$ (%) | $\lvert R_{dc}$ Error$\rvert$ (%) | $\lvert R_{ac}$ Error$\rvert$ (%) | Percent NaN (%) | $\lvert\mu_a$ Error$\rvert$ (%) | $\lvert\mu'_s$ Error$\rvert$ (%) | $\lvert R_{dc}$ Error$\rvert$ (%) | $\lvert R_{ac}$ Error$\rvert$ (%) | Percent NaN (%) | $\lvert\mu_a$ Error$\rvert$ (%) | $\lvert\mu'_s$ Error$\rvert$ (%) | $\lvert R_{dc}$ Error$\rvert$ (%) | $\lvert R_{ac}$ Error$\rvert$ (%) | Percent NaN (%) | $\lvert\mu_a$ Error$\rvert$ (%) | $\lvert\mu'_s$ Error$\rvert$ (%) | $\lvert R_{dc}$ Error$\rvert$ (%) | $\lvert R_{ac}$ Error$\rvert$ (%) | Percent NaN (%) |
| **1** | 49.65 | 34.79 | 10.68 | 23.12 | 0.00 | 93.95 | 73.49 | 8.68 | 61.50 | 0.00 | 74.49 | 45.46 | 9.10 | 49.88 | 0.00 | 90.86 | 81.31 | 2.47 | 81.92 | 0.00 |
| **2** | 39.24 | 1.19 | 21.89 | 3.52 | 0.00 | 49.97 | 16.33 | 14.43 | 15.02 | 0.00 | 38.65 | 5.27 | 8.86 | 5.90 | 0.00 | 29.89 | 11.31 | 4.31 | 15.00 | 0.00 |
| **3** | 60.82 | 4.67 | 24.66 | 1.42 | 0.00 | 42.92 | 0.42 | 18.49 | 1.71 | 0.00 | 23.91 | 6.41 | 4.79 | 6.78 | 0.00 | 27.90 | 1.77 | 5.44 | 1.95 | 0.00 |
| **4** | 57.30 | 6.19 | 24.45 | 13.12 | 0.00 | 54.90 | 1.78 | 18.24 | 4.12 | 0.00 | 14.73 | 5.33 | 5.28 | 6.78 | 0.00 | 12.65 | 3.23 | 3.78 | 4.47 | 0.00 |
| **5** | 88.84 | 86.10 | 2.13 | 43.65 | 0.00 | 84.79 | 56.56 | 16.04 | 38.86 | 0.00 | 53.63 | 42.79 | 5.77 | 41.49 | 0.00 | 79.40 | 56.85 | 8.76 | 53.95 | 0.00 |
| **6** | 28.52 | 7.74 | 14.50 | 2.53 | 0.00 | 58.40 | 37.20 | 10.06 | 35.38 | 0.00 | 19.85 | 19.04 | 0.31 | 23.32 | 0.00 | 28.77 | 22.14 | 2.45 | 28.48 | 0.00 |
| **7** | 40.70 | 7.70 | 19.96 | 1.31 | 0.00 | 32.68 | 3.84 | 15.71 | 1.41 | 0.00 | 5.16 | 2.14 | 3.20 | 3.23 | 0.00 | 6.79 | 1.24 | 3.08 | 2.02 | 0.00 |
| **8** | 29.55 | 1.04 | 17.11 | 7.68 | 0.00 | 22.20 | 7.74 | 15.37 | 11.66 | 0.00 | 5.21 | 6.10 | 4.55 | 7.35 | 0.00 | 4.73 | 14.03 | 3.82 | 16.94 | 0.00 |
| **9** | 28.39 | 12.91 | 26.79 | 4.05 | 0.00 | 21.07 | 76.68 | 56.80 | 68.31 | 0.00 | 63.05 | 83.57 | 11.32 | 83.74 | 0.00 | 67.82 | 86.99 | 9.64 | 82.44 | 0.00 |
| **10** | 27.67 | 5.59 | 20.18 | 9.31 | 0.00 | 8.42 | 21.84 | 13.49 | 21.22 | 0.00 | 6.25 | 2.85 | 2.25 | 2.25 | 0.00 | 14.96 | 15.58 | 0.34 | 18.96 | 0.00 |
| **11** | 2.26 | 1.66 | 0.66 | 0.64 | 0.00 | 3.32 | 0.59 | 2.59 | 0.36 | 0.00 | 2.05 | 0.20 | 1.23 | 0.69 | 0.00 | 4.47 | 0.48 | 2.29 | 1.04 | 0.00 |
| **12** | 0.12 | 6.15 | 6.26 | 7.55 | 0.00 | 2.90 | 3.91 | 5.80 | 5.80 | 0.00 | 4.31 | 0.88 | 2.53 | 1.93 | 0.00 | 2.75 | 2.81 | 2.45 | 3.80 | 0.00 |
| **13** | 14.58 | 29.60 | 17.87 | 13.84 | 81.74 | 25.54 | 72.11 | 45.81 | 52.88 | 0.00 | 20.79 | 38.90 | 15.47 | 38.24 | 0.00 | 49.29 | 77.48 | 17.68 | 69.15 | 0.00 |
| **14** | 11.29 | 2.69 | 15.35 | 12.27 | 0.64 | 9.59 | 18.68 | 39.00 | 29.53 | 0.00 | 10.43 | 12.61 | 1.47 | 13.32 | 0.00 | 26.09 | 22.05 | 2.03 | 25.91 | 0.00 |
| **15** | 10.43 | 27.48 | 22.93 | 25.66 | 0.00 | 8.56 | 0.95 | 9.50 | 4.05 | 0.00 | 0.97 | 2.81 | 1.29 | 2.95 | 0.00 | 7.67 | 2.88 | 2.73 | 2.50 | 0.00 |
| **16** | 28.34 | 5.55 | 37.10 | 24.06 | 0.41 | 14.66 | 4.78 | 12.77 | 3.71 | 0.00 | 8.51 | 8.57 | 0.00 | 6.74 | 0.00 | 11.23 | 9.83 | 0.87 | 10.21 | 0.00 |
| **Average** | **32.36** | **15.07** | **17.66** | **12.11** | | **33.37** | **24.81** | **18.92** | **22.22** | | **22.00** | **17.68** | **4.84** | **18.41** | | **29.08** | **25.62** | **4.51** | **26.17** | |
| **Average subset** | 33.54 | 14.10 | 17.64 | 11.99 | | 33.37 | 24.81 | 18.92 | 22.22 | | 22.00 | 17.68 | 4.84 | 18.41 | | 29.08 | 25.62 | 4.51 | 26.17 | |

**SPF = stHG-High**

| | 395 nm | | | | | 545 nm | | | | | 625 nm | | | | | 850 nm | | | | |
|---|---|---|---|---|---|---|---|---|---|---|---|---|---|---|---|---|---|---|---|---|
| | $\lvert\mu_a$ Error$\rvert$ (%) | $\lvert\mu'_s$ Error$\rvert$ (%) | $\lvert R_{dc}$ Error$\rvert$ (%) | $\lvert R_{ac}$ Error$\rvert$ (%) | Percent NaN (%) | $\lvert\mu_a$ Error$\rvert$ (%) | $\lvert\mu'_s$ Error$\rvert$ (%) | $\lvert R_{dc}$ Error$\rvert$ (%) | $\lvert R_{ac}$ Error$\rvert$ (%) | Percent NaN (%) | $\lvert\mu_a$ Error$\rvert$ (%) | $\lvert\mu'_s$ Error$\rvert$ (%) | $\lvert R_{dc}$ Error$\rvert$ (%) | $\lvert R_{ac}$ Error$\rvert$ (%) | Percent NaN (%) | $\lvert\mu_a$ Error$\rvert$ (%) | $\lvert\mu'_s$ Error$\rvert$ (%) | $\lvert R_{dc}$ Error$\rvert$ (%) | $\lvert R_{ac}$ Error$\rvert$ (%) | Percent NaN (%) |
| **1** | 59.87 | 43.59 | 11.23 | 31.76 | 0.00 | 106.12 | 82.57 | 9.51 | 69.81 | 0.00 | 87.59 | 56.37 | 9.10 | 68.60 | 0.00 | 105.78 | 95.46 | 2.47 | 103.07 | 0.00 |
| **2** | 48.34 | 4.86 | 23.83 | 0.35 | 0.00 | 58.47 | 20.65 | 15.48 | 20.48 | 0.00 | 40.45 | 6.93 | 8.74 | 8.74 | 0.00 | 32.06 | 13.75 | 4.15 | 20.16 | 0.00 |
| **3** | 66.72 | 4.69 | 26.65 | 1.53 | 0.00 | 47.75 | 1.77 | 19.50 | 0.01 | 0.00 | 23.16 | 6.14 | 4.66 | 6.78 | 0.00 | 26.73 | 1.60 | 5.23 | 1.88 | 0.00 |
| **4** | 62.26 | 7.47 | 26.46 | 15.60 | 0.00 | 57.63 | 2.58 | 19.21 | 5.40 | 0.00 | 13.10 | 6.16 | 5.10 | 7.82 | 0.00 | 10.50 | 4.39 | 3.58 | 6.15 | 0.00 |
| **5** | 95.40 | 97.02 | 0.70 | 53.55 | 0.00 | 92.35 | 64.31 | 15.66 | 45.54 | 0.00 | 64.84 | 54.01 | 5.42 | 58.33 | 0.00 | 93.86 | 69.73 | 8.70 | 71.17 | 0.00 |
| **6** | 33.79 | 9.96 | 16.26 | 4.95 | 0.00 | 64.69 | 40.75 | 11.04 | 41.57 | 0.00 | 21.42 | 20.60 | 0.31 | 27.20 | 0.00 | 31.06 | 24.31 | 2.45 | 34.61 | 0.00 |
| **7** | 43.50 | 7.30 | 21.82 | 1.87 | 0.00 | 35.35 | 4.38 | 16.69 | 2.41 | 0.00 | 5.00 | 2.25 | 3.18 | 3.44 | 0.00 | 6.64 | 1.28 | 3.02 | 2.17 | 0.00 |
| **8** | 31.59 | 2.87 | 19.27 | 10.43 | 0.00 | 23.65 | 8.44 | 16.42 | 13.37 | 0.00 | 4.08 | 7.01 | 4.50 | 8.39 | 0.00 | 6.08 | 15.11 | 3.75 | 18.87 | 0.00 |
| **9** | 25.01 | 6.68 | 31.81 | 9.22 | 0.00 | 22.16 | 82.36 | 64.04 | 75.11 | 0.00 | 70.94 | 94.02 | 12.34 | 104.88 | 0.00 | 78.66 | 100.20 | 10.30 | 102.18 | 0.00 |
| **10** | 28.49 | 7.95 | 19.27 | 6.77 | 0.00 | 9.68 | 24.59 | 15.34 | 25.14 | 0.00 | 8.58 | 5.30 | 2.13 | 5.29 | 0.00 | 18.43 | 19.06 | 0.34 | 25.67 | 0.00 |
| **11** | 2.80 | 2.08 | 0.82 | 0.86 | 0.00 | 3.16 | 0.47 | 2.59 | 0.36 | 0.00 | 2.08 | 0.18 | 1.23 | 0.69 | 0.00 | 4.49 | 0.46 | 2.29 | 1.04 | 0.00 |
| **12** | 1.29 | 8.48 | 7.45 | 9.92 | 0.00 | 1.30 | 5.99 | 6.40 | 8.39 | 0.00 | 3.15 | 1.98 | 2.53 | 2.91 | 0.00 | 1.09 | 4.37 | 2.44 | 5.75 | 0.00 |
| **13** | 9.85 | 19.56 | 11.75 | 8.98 | 59.08 | 13.94 | 65.28 | 58.98 | 57.99 | 0.00 | 28.39 | 49.96 | 17.77 | 52.81 | 0.00 | 61.61 | 93.97 | 19.10 | 87.16 | 0.00 |
| **14** | 7.56 | 2.31 | 11.74 | 9.35 | 0.10 | 12.84 | 17.95 | 46.35 | 32.19 | 0.00 | 12.29 | 14.77 | 1.67 | 16.76 | 0.00 | 29.26 | 25.19 | 2.01 | 32.94 | 0.00 |
| **15** | 13.91 | 28.13 | 20.64 | 24.79 | 0.00 | 12.00 | 2.69 | 12.67 | 4.03 | 0.00 | 1.00 | 3.00 | 1.40 | 3.31 | 0.00 | 7.88 | 3.10 | 2.72 | 3.02 | 0.00 |
| **16** | 30.26 | 7.85 | 39.01 | 23.34 | 0.28 | 18.85 | 8.37 | 14.77 | 1.39 | 0.00 | 9.43 | 9.49 | 0.00 | 7.70 | 0.00 | 12.36 | 10.99 | 0.86 | 12.00 | 0.00 |
| **Average** | **35.04** | **16.30** | **18.04** | **13.33** | | **36.25** | **27.07** | **21.54** | **25.20** | | **24.72** | **21.14** | **5.00** | **23.98** | | **32.90** | **30.19** | **4.59** | **32.99** | |
| **Average subset** | 36.72 | 16.08 | 18.46 | 13.62 | | 36.25 | 27.07 | 21.54 | 25.20 | | 24.72 | 21.14 | 5.00 | 23.98 | | 32.90 | 30.19 | 4.59 | 32.99 | |

## Table S2. Absolute percentage errors of SFDI using a phantom reference and cross-polarization for different scattering phase functions.

**SPF = ttHG**

| | 395 nm | | | | | 545 nm | | | | | 625 nm | | | | | 850 nm | | | | |
|---|---|---|---|---|---|---|---|---|---|---|---|---|---|---|---|---|---|---|---|---|
| | $\lvert\mu_a$ Error$\rvert$ (%) | $\lvert\mu'_s$ Error$\rvert$ (%) | $\lvert R_{dc}$ Error$\rvert$ (%) | $\lvert R_{ac}$ Error$\rvert$ (%) | Percent NaN (%) | $\lvert\mu_a$ Error$\rvert$ (%) | $\lvert\mu'_s$ Error$\rvert$ (%) | $\lvert R_{dc}$ Error$\rvert$ (%) | $\lvert R_{ac}$ Error$\rvert$ (%) | Percent NaN (%) | $\lvert\mu_a$ Error$\rvert$ (%) | $\lvert\mu'_s$ Error$\rvert$ (%) | $\lvert R_{dc}$ Error$\rvert$ (%) | $\lvert R_{ac}$ Error$\rvert$ (%) | Percent NaN (%) | $\lvert\mu_a$ Error$\rvert$ (%) | $\lvert\mu'_s$ Error$\rvert$ (%) | $\lvert R_{dc}$ Error$\rvert$ (%) | $\lvert R_{ac}$ Error$\rvert$ (%) | Percent NaN (%) |
| **1** | 48.92 | 51.60 | 4.46 | 36.68 | 0.00 | 37.55 | 29.52 | 8.39 | 24.44 | 0.00 | 62.11 | 67.07 | 6.92 | 58.38 | 0.00 | 64.97 | 66.43 | 2.60 | 61.04 | 0.00 |
| **2** | 42.81 | 29.06 | 14.56 | 18.04 | 0.00 | 50.40 | 37.30 | 13.09 | 28.40 | 0.00 | 14.72 | 19.81 | 1.91 | 16.89 | 0.00 | 30.02 | 26.63 | 1.31 | 21.28 | 0.00 |
| **3** | 14.09 | 15.68 | 17.36 | 15.24 | 0.00 | 30.08 | 17.40 | 8.63 | 11.91 | 0.00 | 0.91 | 8.91 | 2.40 | 8.02 | 0.00 | 0.61 | 1.15 | 0.44 | 1.03 | 0.00 |
| **4** | 27.57 | 18.32 | 23.61 | 19.45 | 0.00 | 29.78 | 2.58 | 14.92 | 3.34 | 0.00 | 2.86 | 6.76 | 2.59 | 6.11 | 0.00 | 2.40 | 10.61 | 1.93 | 9.59 | 0.00 |
| **5** | 16.41 | 44.99 | 32.29 | 36.91 | 0.00 | 47.12 | 62.96 | 25.75 | 55.15 | 0.00 | 62.37 | 72.37 | 19.85 | 61.39 | 0.00 | 68.67 | 76.85 | 17.26 | 69.71 | 0.00 |
| **6** | 31.05 | 14.50 | 16.59 | 4.27 | 0.00 | 20.98 | 3.92 | 12.91 | 1.16 | 0.00 | 9.08 | 3.24 | 3.09 | 1.91 | 0.00 | 9.41 | 10.37 | 0.47 | 7.92 | 0.00 |
| **7** | 4.75 | 10.90 | 10.88 | 10.48 | 0.00 | 19.92 | 10.74 | 6.66 | 6.34 | 0.00 | 4.17 | 1.26 | 1.33 | 0.65 | 0.00 | 1.65 | 2.43 | 0.27 | 2.06 | 0.00 |
| **8** | 12.23 | 28.52 | 24.32 | 27.75 | 0.00 | 21.29 | 1.57 | 13.77 | 3.41 | 0.00 | 6.74 | 9.49 | 1.04 | 7.08 | 0.00 | 4.86 | 3.45 | 0.47 | 2.69 | 0.00 |
| **9** | 56.00 | 67.88 | 25.83 | 44.00 | 0.00 | 0.07 | 21.38 | 20.39 | 20.58 | 0.00 | 47.31 | 58.34 | 16.73 | 45.27 | 0.00 | 62.67 | 70.28 | 15.70 | 61.03 | 0.00 |
| **10** | 2.27 | 37.51 | 33.24 | 33.22 | 0.00 | 36.75 | 43.57 | 9.40 | 28.32 | 0.00 | 27.17 | 37.73 | 9.52 | 28.37 | 0.00 | 41.64 | 45.96 | 4.21 | 35.05 | 0.00 |
| **11** | 4.92 | 5.41 | 0.38 | 1.15 | 0.00 | 11.27 | 10.79 | 0.35 | 4.67 | 0.00 | 1.97 | 2.64 | 0.39 | 1.86 | 0.00 | 5.02 | 2.93 | 0.88 | 2.07 | 0.00 |
| **12** | 9.54 | 20.81 | 7.90 | 10.37 | 0.05 | 32.72 | 43.40 | 5.68 | 22.58 | 0.00 | 21.63 | 24.75 | 1.24 | 14.54 | 0.00 | 33.23 | 35.09 | 0.62 | 25.43 | 0.00 |
| **13** | | | 75.31 | 71.77 | 100.00 | | | 58.46 | 48.76 | 100.00 | 67.50 | 76.74 | 24.65 | 61.74 | 0.00 | 76.98 | 81.41 | 15.71 | 74.24 | 0.00 |
| **14** | 0.80 | 56.65 | 54.59 | 49.43 | 99.93 | 34.72 | 1.04 | 23.57 | 18.25 | 1.76 | 8.90 | 15.83 | 5.03 | 11.71 | 0.00 | 11.31 | 19.99 | 5.87 | 15.05 | 0.00 |
| **15** | 0.42 | 56.42 | 52.94 | 47.86 | 99.97 | 113.80 | 49.51 | 27.55 | 16.11 | 6.71 | 6.20 | 2.95 | 5.45 | 4.34 | 0.00 | 7.59 | 7.14 | 8.06 | 7.28 | 0.00 |
| **16** | 49.07 | 71.86 | 13.72 | 16.75 | 10.30 | 278.04 | 191.21 | 19.67 | 1.30 | 62.92 | 15.51 | 11.21 | 2.27 | 2.20 | 0.32 | 16.88 | 9.94 | 3.27 | 4.06 | 0.32 |
| **Average** | **21.39** | **35.34** | **25.50** | **27.71** | | **50.97** | **35.13** | **16.82** | **18.42** | | **22.45** | **26.19** | **6.53** | **20.65** | | **27.37** | **29.42** | **4.94** | **24.97** | |
| **Average subset** | 22.55 | 28.77 | 17.62 | 21.46 | | 28.67 | 22.01 | 12.58 | 17.58 | | 22.45 | 26.19 | 6.53 | 20.65 | | 27.37 | 29.42 | 4.94 | 24.97 | |

**SPF = stHG-Low**

| | 395 nm | | | | | 545 nm | | | | | 625 nm | | | | | 850 nm | | | | |
|---|---|---|---|---|---|---|---|---|---|---|---|---|---|---|---|---|---|---|---|---|
| | $\lvert\mu_a$ Error$\rvert$ (%) | $\lvert\mu'_s$ Error$\rvert$ (%) | $\lvert R_{dc}$ Error$\rvert$ (%) | $\lvert R_{ac}$ Error$\rvert$ (%) | Percent NaN (%) | $\lvert\mu_a$ Error$\rvert$ (%) | $\lvert\mu'_s$ Error$\rvert$ (%) | $\lvert R_{dc}$ Error$\rvert$ (%) | $\lvert R_{ac}$ Error$\rvert$ (%) | Percent NaN (%) | $\lvert\mu_a$ Error$\rvert$ (%) | $\lvert\mu'_s$ Error$\rvert$ (%) | $\lvert R_{dc}$ Error$\rvert$ (%) | $\lvert R_{ac}$ Error$\rvert$ (%) | Percent NaN (%) | $\lvert\mu_a$ Error$\rvert$ (%) | $\lvert\mu'_s$ Error$\rvert$ (%) | $\lvert R_{dc}$ Error$\rvert$ (%) | $\lvert R_{ac}$ Error$\rvert$ (%) | Percent NaN (%) |
| **1** | 1.74 | 9.74 | 8.70 | 9.78 | 0.00 | 12.15 | 10.18 | 1.49 | 8.69 | 0.00 | 11.30 | 23.97 | 7.87 | 23.57 | 0.00 | 29.45 | 32.65 | 2.50 | 30.07 | 0.00 |
| **2** | 10.69 | 2.73 | 9.23 | 4.59 | 0.00 | 4.25 | 10.01 | 3.58 | 9.99 | 0.00 | 13.30 | 2.03 | 4.64 | 3.10 | 0.00 | 1.67 | 2.64 | 0.25 | 3.39 | 0.00 |
| **3** | 32.84 | 11.79 | 10.29 | 11.14 | 0.00 | 10.23 | 5.43 | 8.17 | 6.88 | 0.00 | 8.51 | 6.70 | 0.48 | 7.96 | 0.00 | 5.85 | 0.34 | 1.21 | 0.37 | 0.00 |
| **4** | 18.57 | 1.50 | 7.57 | 0.05 | 0.00 | 7.75 | 3.80 | 4.57 | 5.14 | 0.00 | 0.84 | 2.59 | 0.45 | 2.91 | 0.00 | 2.15 | 1.21 | 0.28 | 1.51 | 0.00 |
| **5** | 24.26 | 4.94 | 18.64 | 4.36 | 0.00 | 22.50 | 39.45 | 22.85 | 36.12 | 0.00 | 17.48 | 31.13 | 13.86 | 30.53 | 0.00 | 37.52 | 49.82 | 14.06 | 46.91 | 0.00 |
| **6** | 7.87 | 5.04 | 2.26 | 4.35 | 0.00 | 24.42 | 24.09 | 0.19 | 23.92 | 0.00 | 8.98 | 13.63 | 2.22 | 17.34 | 0.00 | 13.07 | 11.56 | 0.60 | 14.79 | 0.00 |
| **7** | 24.09 | 9.15 | 9.92 | 4.83 | 0.00 | 8.77 | 2.54 | 7.20 | 3.89 | 0.00 | 0.80 | 1.94 | 0.48 | 2.26 | 0.00 | 0.74 | 2.20 | 1.15 | 2.95 | 0.00 |
| **8** | 13.93 | 7.81 | 3.63 | 6.40 | 0.00 | 0.33 | 5.65 | 3.42 | 7.31 | 0.00 | 1.07 | 2.06 | 1.23 | 2.40 | 0.00 | 5.70 | 9.56 | 1.52 | 11.35 | 0.00 |
| **9** | 37.48 | 40.06 | 5.27 | 25.80 | 0.00 | 4.09 | 11.68 | 8.61 | 9.70 | 0.00 | 3.69 | 1.84 | 4.97 | 2.63 | 0.00 | 30.68 | 34.92 | 5.41 | 31.64 | 0.00 |
| **10** | 13.51 | 13.94 | 27.89 | 22.97 | 0.00 | 19.84 | 17.84 | 2.77 | 13.26 | 0.00 | 3.74 | 12.17 | 6.31 | 14.76 | 0.00 | 10.36 | 13.55 | 2.15 | 15.76 | 0.00 |
| **11** | 2.48 | 1.12 | 1.54 | 0.20 | 0.00 | 5.78 | 5.39 | 0.35 | 4.67 | 0.00 | 1.16 | 1.79 | 0.39 | 1.86 | 0.00 | 3.77 | 1.84 | 0.88 | 2.07 | 0.00 |
| **12** | 3.19 | 5.42 | 2.28 | 4.66 | 0.00 | 8.59 | 7.68 | 0.73 | 6.86 | 0.00 | 6.54 | 6.73 | 0.13 | 5.49 | 0.00 | 9.36 | 9.79 | 0.19 | 10.75 | 0.00 |
| **13** | | | 61.54 | 59.24 | 100.00 | 16.58 | 36.05 | 27.33 | 29.79 | 0.40 | 35.84 | 39.35 | 5.74 | 34.29 | 0.00 | 59.30 | 59.13 | 0.37 | 53.31 | 0.00 |
| **14** | 8.38 | 29.48 | 41.01 | 38.31 | 2.94 | 18.97 | 13.52 | 8.28 | 3.82 | 0.00 | 6.43 | 5.59 | 0.56 | 5.49 | 0.00 | 14.41 | 9.01 | 3.03 | 10.06 | 0.00 |
| **15** | 0.25 | 37.12 | 43.22 | 43.33 | 0.25 | 4.71 | 13.47 | 10.72 | 13.51 | 0.00 | 2.65 | 0.91 | 2.40 | 1.95 | 0.00 | 7.32 | 2.72 | 5.88 | 4.63 | 0.00 |
| **16** | 23.74 | 7.62 | 24.80 | 14.89 | 0.00 | 1.09 | 8.09 | 7.96 | 9.50 | 0.00 | 2.66 | 0.18 | 2.33 | 6.20 | 0.09 | 0.61 | 4.57 | 2.48 | 7.94 | 0.12 |
| **Average** | **14.87** | **12.50** | **17.36** | **15.93** | | **10.63** | **13.43** | **7.39** | **12.07** | | **7.81** | **9.54** | **3.38** | **10.17** | | **14.50** | **15.34** | **2.62** | **15.47** | |
| **Average subset** | 15.33 | 11.28 | 12.52 | 11.24 | | 10.63 | 13.43 | 7.39 | 12.07 | | 7.81 | 9.54 | 3.38 | 10.17 | | 14.50 | 15.34 | 2.62 | 15.47 | |

**SPF = stHG-High**

| | 395 nm | | | | | 545 nm | | | | | 625 nm | | | | | 850 nm | | | | |
|---|---|---|---|---|---|---|---|---|---|---|---|---|---|---|---|---|---|---|---|---|
| | $\lvert\mu_a$ Error$\rvert$ (%) | $\lvert\mu'_s$ Error$\rvert$ (%) | $\lvert R_{dc}$ Error$\rvert$ (%) | $\lvert R_{ac}$ Error$\rvert$ (%) | Percent NaN (%) | $\lvert\mu_a$ Error$\rvert$ (%) | $\lvert\mu'_s$ Error$\rvert$ (%) | $\lvert R_{dc}$ Error$\rvert$ (%) | $\lvert R_{ac}$ Error$\rvert$ (%) | Percent NaN (%) | $\lvert\mu_a$ Error$\rvert$ (%) | $\lvert\mu'_s$ Error$\rvert$ (%) | $\lvert R_{dc}$ Error$\rvert$ (%) | $\lvert R_{ac}$ Error$\rvert$ (%) | Percent NaN (%) | $\lvert\mu_a$ Error$\rvert$ (%) | $\lvert\mu'_s$ Error$\rvert$ (%) | $\lvert R_{dc}$ Error$\rvert$ (%) | $\lvert R_{ac}$ Error$\rvert$ (%) | Percent NaN (%) |
| **1** | 6.19 | 1.78 | 4.58 | 0.45 | 0.00 | 19.97 | 16.57 | 2.38 | 14.28 | 0.00 | 0.47 | 13.92 | 7.87 | 14.02 | 0.00 | 21.14 | 24.77 | 2.51 | 21.94 | 0.00 |
| **2** | 13.40 | 3.06 | 6.92 | 2.38 | 0.00 | 2.79 | 5.35 | 4.77 | 5.72 | 0.00 | 15.20 | 0.05 | 4.51 | 0.50 | 0.00 | 0.99 | 0.62 | 0.07 | 0.96 | 0.00 |
| **3** | 29.18 | 12.76 | 8.16 | 14.31 | 0.00 | 14.75 | 3.65 | 9.31 | 5.28 | 0.00 | 7.77 | 6.42 | 0.34 | 7.97 | 0.00 | 4.79 | 0.26 | 0.99 | 0.31 | 0.00 |
| **4** | 12.92 | 1.09 | 5.40 | 0.18 | 0.00 | 10.18 | 4.38 | 5.70 | 6.41 | 0.00 | 2.46 | 3.53 | 0.26 | 3.99 | 0.00 | 4.29 | 2.49 | 0.50 | 3.24 | 0.00 |
| **5** | 28.56 | 16.28 | 11.97 | 5.31 | 0.00 | 19.51 | 36.45 | 22.51 | 33.05 | 0.00 | 6.27 | 21.28 | 13.54 | 22.27 | 0.00 | 29.77 | 43.49 | 14.00 | 40.98 | 0.00 |
| **6** | 8.39 | 9.18 | 0.66 | 10.34 | 0.00 | 30.53 | 28.24 | 1.27 | 29.59 | 0.00 | 10.68 | 15.40 | 2.22 | 21.02 | 0.00 | 15.90 | 14.36 | 0.60 | 20.27 | 0.00 |
| **7** | 20.71 | 9.78 | 7.47 | 7.42 | 0.00 | 11.78 | 1.46 | 8.28 | 2.94 | 0.00 | 0.97 | 2.06 | 0.46 | 2.47 | 0.00 | 0.65 | 2.18 | 1.10 | 3.10 | 0.00 |
| **8** | 8.44 | 6.33 | 1.30 | 6.34 | 0.00 | 1.60 | 6.47 | 4.62 | 9.11 | 0.00 | 0.18 | 3.14 | 1.17 | 3.49 | 0.00 | 7.30 | 10.92 | 1.45 | 13.41 | 0.00 |
| **9** | 35.64 | 33.73 | 3.55 | 19.60 | 0.00 | 4.68 | 16.10 | 13.63 | 14.13 | 0.00 | 14.50 | 9.54 | 4.10 | 8.57 | 0.00 | 22.54 | 26.73 | 4.84 | 24.24 | 0.00 |
| **10** | 11.82 | 10.01 | 23.30 | 18.40 | 0.00 | 17.36 | 14.15 | 4.45 | 10.46 | 0.00 | 0.61 | 9.11 | 6.19 | 12.22 | 0.00 | 5.23 | 8.60 | 2.14 | 11.01 | 0.00 |
| **11** | 7.34 | 1.71 | 6.95 | 3.00 | 0.00 | 5.25 | 4.86 | 0.35 | 4.67 | 0.00 | 1.07 | 1.71 | 0.39 | 1.86 | 0.00 | 3.66 | 1.72 | 0.88 | 2.07 | 0.00 |
| **12** | 9.27 | 7.98 | 1.45 | 4.31 | 0.00 | 5.96 | 4.32 | 1.36 | 3.92 | 0.00 | 5.09 | 5.28 | 0.13 | 4.43 | 0.00 | 6.92 | 7.39 | 0.21 | 8.51 | 0.00 |
| **13** | | | 56.55 | 55.64 | 100.00 | 25.02 | 37.86 | 20.77 | 27.44 | 0.08 | 27.72 | 30.31 | 3.85 | 27.37 | 0.00 | 55.25 | 54.52 | 1.59 | 48.34 | 0.00 |
| **14** | 0.60 | 29.30 | 35.33 | 34.30 | 0.84 | 21.33 | 12.78 | 14.01 | 1.85 | 0.00 | 8.61 | 8.04 | 0.37 | 8.69 | 0.00 | 18.42 | 12.88 | 3.01 | 16.21 | 0.00 |
| **15** | 10.94 | 39.18 | 38.52 | 40.83 | 0.00 | 8.38 | 14.54 | 8.13 | 13.53 | 0.00 | 2.84 | 0.54 | 2.29 | 1.61 | 0.00 | 7.91 | 2.14 | 5.87 | 4.15 | 0.00 |
| **16** | 30.23 | 11.31 | 33.06 | 17.62 | 0.00 | 7.04 | 12.10 | 6.33 | 11.52 | 0.00 | 1.22 | 1.65 | 2.33 | 7.16 | 0.06 | 2.58 | 6.59 | 2.49 | 9.78 | 0.11 |
| **Average** | **15.58** | **12.90** | **15.32** | **15.03** | | **12.88** | **13.71** | **7.99** | **12.12** | | **6.60** | **8.25** | **3.13** | **9.23** | | **12.96** | **13.73** | **2.64** | **14.28** | |
| **Average subset** | 15.58 | 12.90 | 12.57 | 12.32 | | 12.88 | 13.71 | 7.99 | 12.12 | | 6.60 | 8.25 | 3.13 | 9.23 | | 12.96 | 13.73 | 2.64 | 14.28 | |
